# Genome-wide association study of self-reported food reactions in Japanese identifies shrimp and peach specific loci in the *HLA-DR*/*DQ* gene region

**DOI:** 10.1038/s41598-017-18241-w

**Published:** 2018-01-18

**Authors:** Seik-Soon Khor, Ryoko Morino, Kazuyuki Nakazono, Shigeo Kamitsuji, Masanori Akita, Maiko Kawajiri, Tatsuya Yamasaki, Azusa Kami, Yuria Hoshi, Asami Tada, Kenichi Ishikawa, Maaya Hine, Miki Kobayashi, Nami Kurume, Naoyuki Kamatani, Katsushi Tokunaga, Todd A. Johnson

**Affiliations:** 1 Department of Human Genetics, Graduate School of Medicine, The University of Tokyo, Tokyo, 113-0033 Japan; 2 EverGene Ltd., Shinjuku-ku, Tokyo, 163-1435 Japan; 3 StaGen Co., Ltd., Taito-ku, Tokyo, 111-0051 Japan; 4 Life Science Group, Healthcare Division, Department of Healthcare Business, MTI Ltd., Shinjuku-ku, Tokyo, 163-1435 Japan; 5 LunaLuna Division, Department of Healthcare Business, MTI Ltd., Shinjuku-ku, Tokyo, 163-1435 Japan

## Abstract

Food allergy is an increasingly important health problem in the world. Several genome-wide association studies (GWAS) focused on European ancestry samples have identified food allergy-specific loci in the HLA class II region. We conducted GWAS of self-reported reactivity with common foods using the data from 11011 Japanese women and identified shrimp and peach allergy-specific loci in the *HLA-DR/DQ* gene region tagged by rs74995702 (*P* = 6.30 × 10^−17^, *OR* = 1.91) and rs28359884 (*P* = 2.3 × 10^−12^, *OR* = 1.80), respectively. After HLA imputation using a Japanese population-specific reference, the most strongly associated haplotype was HLA-DRB1*04:05-HLA-DQB1*04:01 for shrimp allergy (*P* = 3.92 × 10^−19^, *OR* = 1.99) and HLA-DRB1*09:01-HLA-DQB1*03:03 for peach allergy (*P* = 1.15 × 10^−7^, *OR* = 1.68). Additionally, both allergies’ associated variants were eQTLs for several HLA genes, with *HLA-DQA*2 the single eQTL gene shared between the two traits. Our study suggests that allergy to certain foods may be related to genetic differences that tag both HLA alleles having particular epitope binding specificities as well as variants modulating expression of particular HLA genes. Investigating this further could increase our understanding of food allergy aetiology and potentially lead to better therapeutic strategies for allergen immunotherapies.

## Introduction

Asthma has been traditionally known to be the first wave of the allergy epidemic affecting the lives of millions and is the third leading cause for hospitalization in the United States among people less than 18 years of age^1^. Food allergy appears to be increasing in its prevalence, with 5% of adults and 8% of children estimated to be affected^2^, and it is now commonly referred to as the second wave of the allergy epidemic^3^. In general, individuals during childhood are at higher risk for food allergies to wheat, soy, milk and egg, but food allergies to tree nuts, peanuts, fish and shellfish normally tend to persist throughout adulthood^2^. In addition, recent studies have shown that sensitization to food can occur in adulthood^4^, although serum IgE reactivity with specific foods appears to differ by geographic region, presumably due to differential consumption of particular foods or exposure to environmental allergens^4–7^.

Food allergy related disorders include acute conditions with the potential to be fatal, along with a number of chronic diseases that mainly occur in the epidermis and gastrointestinal tract^8^. Such disorders can roughly be divided into classical IgE-mediated food allergies or those mediated by other antibody isotypes such as IgA and IgG^9^. In addition, food allergy diagnosis can be complicated because detection of food-specific IgE does not always correlate with clinically defined allergy^10^, and there are a number of disorders for which symptoms may appear to be similar to food allergy. For example, dark-meat fish can contain histamine-like chemicals that cause a toxic reaction known as scombroid poisoning^11^, and consumption of spicy foods may result in rhinorrhea, a situation known as gustatory rhinitis, which mimics aspects of food allergy^12^. In addition, individuals with food intolerance may misascribe their symptoms as “allergies”. For example, one study using self-reported food reactions found that of subjects reporting reactivity to milk and wheat, 85% and 94% could be ascribed to lactose and gluten intolerance, respectively^5^.

In a nationwide survey of Japanese physicians for immediate-type food allergies across all age groups, the percentage of 2,954 patients that reacted with causative foods were: hen’s eggs (39.0%), cow’s milk (21.8%), wheat (11.7%) peanuts (5.1%), fruit (4%), fish roe (3.7%), crustaceans (3.4%), nuts (2.3%), buckwheat (2.2%), fish (2.1%), and other foods (4.6%)^13^. In contrast, foods that made up greater than 5% of either new-onset food allergies (NOFA) or patient visits due to accidental ingestion (AIFA) in adults (≥20 years old) were limited to wheat (NOFA = 38.0%; AIFA = 34.0%), crustaceans (NOFA = 10.0%; AIFA = 22.0%), buckwheat (NOFA = NA; AIFA = 10.0%), fish (NOFA = 13.0%; AIFA = 8.0%), and fruit (NOFA = 7.0%; AIFA = 8.0%). The levels of wheat-related NOFA and AIFA in adults was around three times greater than that seen across the five lower age ranges that were tabulated and may be related to recent reports of wheat allergy and wheat-dependent exercise-induced urticaria/anaphylaxis attributed to use of soap and other cosmetic products containing hydrolysed wheat protein^14^. Based on such surveys, the food labelling system for allergenic food ingredients in Japan started on April 1, 2002 designated egg, milk, wheat, buckwheat and peanuts as mandatory, and the labelling of shrimp and crab was required from 2008^15^. Labelling of an additional twenty foods is recommended by the Consumer Affairs Agency^13^.

The human major histocompatibility complex (MHC) region encodes the human leukocyte antigen (HLA) genes, among which HLA class II molecules (*HLA-DR*, *-DQ* and *-DP*) have especially been reported to be involved in food allergy responses. *HLA* involvement in the IgE response was first described when Levine *et al*. reported that *HLA* class I haplotypes triggered IgE response to allergen derived from ragweed (*Ambrosia artemisiaefolia*)^16^. Subsequently, other *HLA* associations with allergens were reported, including for rye grass (*Lol p* I, *Lol p* II, *Lol p* III)^17,18^, American feverfew, and birch pollen (*Bet v* I) with *HLA-DR3*^19^. With the advancement of methods for genotyping, genome-wide association studies (GWASs) have been used to identify susceptibility genes for allergic diseases such as asthma^20–22^ and atopic dermatitis^23–25^. Besides that, association of *HLA-DR* and *HLA-DQ* were confirmed for several food allergies including peanut^26–28^ and asparaginase (a kind of enzyme used in food manufacturing)^29^.

Foods generally contain multiple proteins to which individuals may react differently due to exposure of the food to heat (cooking), acid, and proteases (gastrointestinal tract), and most of the identified relevant food allergens are water-soluble glycoproteins^30–33^. In addition, antibodies may bind to either linear or conformational epitopes within a protein’s structure, with the former representing short runs of amino acids within the primary protein structure, and the latter representing epitopes within the three-dimensional structure of the protein. Previous studies have shown that the repertoire of antibodies binding to multiple epitopes within food allergens may correlate with disease status^34^, and IgE that recognizes the tertiary allergen structure is often associated with transient childhood allergy while linear epitope recognition often results in persistence allergy into adulthood^34,35^.

In this study, we performed a comprehensive analysis of self-reported reactivity with 27 different foods from fruits, vegetables, grains, meats, seafood, and dairy product food groups in a Japanese population sample.

## Results

### GWAS of self-reported food reactions in the Japanese population

Samples for this study were collected as part of an on-going effort of the Japanese company MTI and its subsidiary EverGene (EG) to better understand and improve women’s health through research use of its Luna Luna family of women’s healthcare related web-site and smartphone app. DNA from 11379 female subjects was collected in two study stages (LL01 = 5751, LL02 = 5628), and subjects were queried via questionnaire for putative allergic sensitivity to twenty-seven different foods from fruits, legumes/grains, nuts, vegetables/mushrooms, eggs/dairy, meats, fish, and shellfish product food groups (Supplementary Table S1). DNA samples were genotyped using a custom Affymetrix Axiom genotyping array based on the company’s CHB-1 product (SNP ct. = 607857; see Methods), and using 536506 QC + variants, we analysed LL01/LL02 samples for population structure and for the presence of duplicated samples via principal component analysis (PCA; Supplementary Fig. S1) and identity-by-descent (IBD) analysis (see Methods); 11011 QC + subjects also answered the food allergy questions and were used in our analysis.

Preliminary GWAS scans were performed across the 27 foods, and then, due to noise and a lack of signals for foods with low case sample counts, final GWAS analyses were run across seven foods for which the number of cases were deemed sufficient (>100 cases in both the LL01 and LL02 sample sets; kiwi, peach, Chinese yam, eggs, mackerel, crab, and shrimp in Supplementary Table S1). Evaluation of genome-wide inflation of test statistics showed that *λ*_*GC*_ values for genotyped variants ranged from 0.9889 to 1.0073 across the seven foods, which suggested that there was negligible inflation of the test statistics after adjustment for the top two PCs (Supplementary Fig. S2).

**Figure 1:**
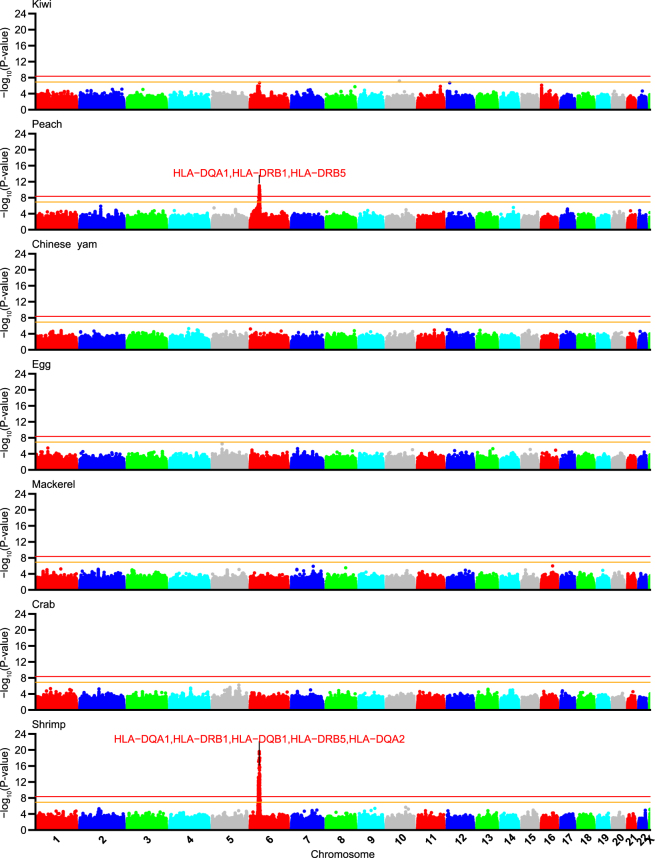
Manhattan plots of meta-analysis *P*-values for food reactivity GWAS. Manhattan plots of −log_10_(*P*_*meta*_) for GWAS of self-reported food reactions to seven foods with >100 samples in both LL01 and LL02 sample-sets. Summary statistics based imputed meta-analysis data was filtered for MAF ≥ 0.01 and DISTMIX INFO > 0.8. Top shrimp and peach association peaks in the HLA region were labelled with up to five annotated genes from Supplementary Worksheet S1 for variants with *r*^2^ > 0.8 to the top SNP in peach (rs28359884) or shrimp (rs74995702).

Since neither LL01 or LL02 was originally considered as a traditional discovery sampleset with the other being a “replication” set, we employed a bi-directional “discovery-evaluation” GWAS strategy that was recently described (see Methods; Flow-chart: Supplementary Fig. S3)^36^. We then combined the two samplesets in a meta-analysis, and based on a previous estimate of the effective independent SNP count (M_E_) for a similarly sized array platform and the JPT population^37^, we called SNPs that achieved a single GWAS P-value cut-off of *P*_*meta*_ < 1.2 × 10^−7^ (0.05/411,521 SNPs) as nominal associations and those achieving a multiple testing adjusted cut-off of *P*_*meta*_ < 4.4 × 10^−9^ (1.2 × 10^−7^/27 foods) as strong associations. Two foods were identified with SNPs achieving the latter threshold (peach and shrimp; Table 1). To check for potential signals that may have escaped detection by using only genotyped data for identifying association signals, we also performed a genome-wide summary statistics based imputation using DISTMIX (see Methods) and plotted the results as Manhattan plots in Fig. 1. No additional signals achieving the significance cutoff were identified in any of the seven foods as compared to the original analysis that used genotyped data.

**Table 1:** Summary of food-allergy association signals.

Food	Chr.	Signal range (*r*^2^ > 0.5)	SNP ct. (*r*^2^ > 0.8)	Top SNP rsid	Effect/Other alleles	Top SNP *P*-value			*OR*		
						LL01	LL02	Meta.	LL01	LL02	Meta.
Peach	6	32.55–32.68 Mb	46	rs28359884	C/A	8.4 × 10^−6^	4.7 × 10^−8^	2.3 × 10^−12^	1.70[1.34–2.14]	1.91[1.51–2.40]	1.80[1.53–2.12]
Shrimp	6	32.15–32.68 Mb	30	rs74995702	G/A	1.6 × 10^−14^	5.3 × 10^−5^	6.3 × 10^−17^	2.27[1.84–2.80]	1.58[1.27–1.97]	1.91[1.64–2.23]

Odds-ratio is oriented to the allele that had increasing effect and formatted as OR[95% confidence interval].

### Associations with peach and shrimp allergy in the *HLA* region

**Figure 2:**
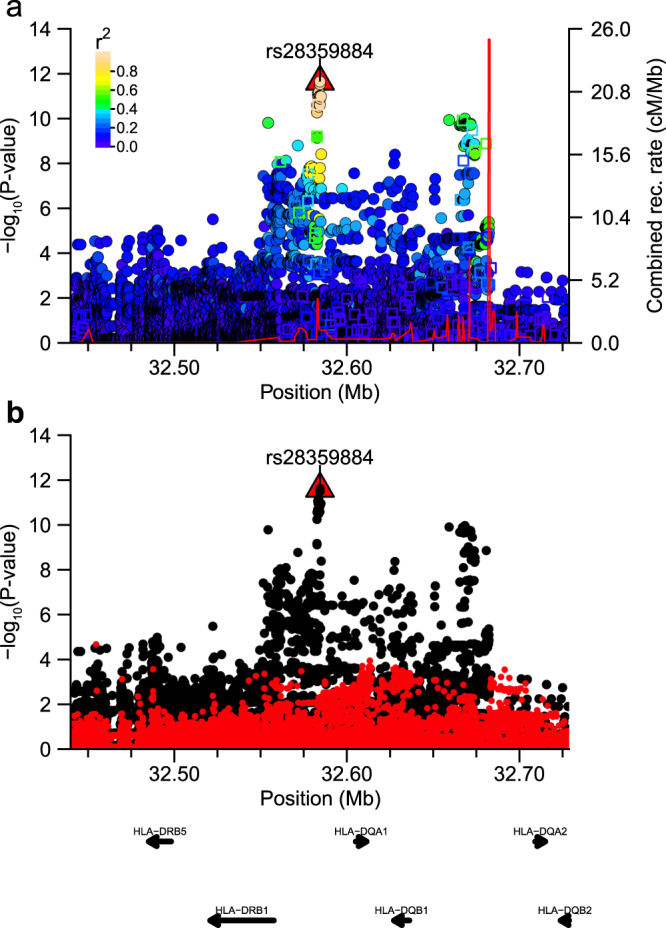
Regional plot around peach association signal. Plot of association statistics around rs28359884. Panel (a) points coloured by LD *r*^*2*^ to the top SNP. Imputed variants were plotted as circles and genotyped data as squares. The red line shows the estimated recombination rate in 1000 G JPT samples. Panel (b) unadjusted statistics plotted as black dots and statistics after conditioning the logistic regression on rs28359884 plotted as red dots.

**Figure 3:**
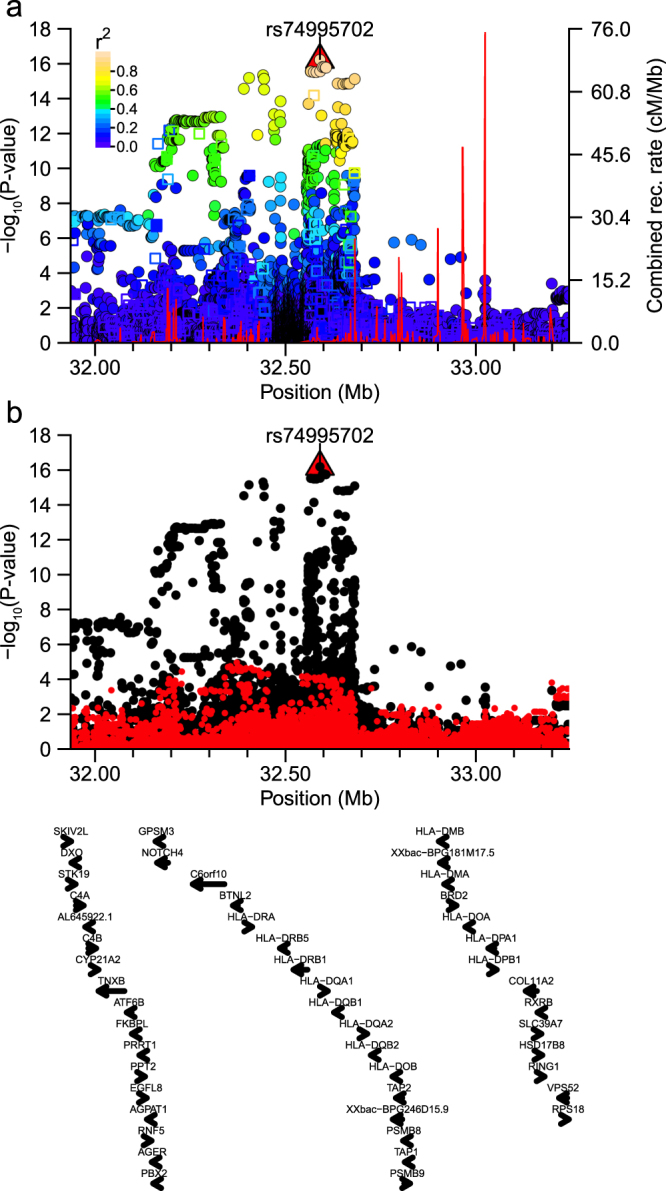
Regional plot around shrimp association signal. Plot of association statistics around rs74995702. Panel (a) points coloured by LD *r*^*2*^ to the top SNP. Imputed variants were plotted as circles and genotyped data as squares. The red line shows the estimated recombination rate in 1000 G JPT samples. Panel (b) unadjusted statistics plotted as black dots and statistics after conditioning the logistic regression on rs74995702 plotted as red dots.

Within both sets of peach and shrimp allergy associated variants, we identified a single cluster of neighbouring SNPs in the *HLA-DR*/*HLA-DQ* region of chromosome six (Fig. 1). To more accurately examine the underlying signals, we performed regional genotype imputation using 1000 Genomes Phase 3 reference haplotypes and identified rs28359884 and rs74995702 as the top SNP in the fine-mapped peach (Fig. 2a) and shrimp (Fig. 3a) allergy association signals, respectively; we found no residual significant signal after conditioning the regression analyses for each top imputed variant (Table 1; Fig. 2b and Fig. 3b).

We annotated variants that were in linkage disequilibrium (LD) to the top SNP in each signal using HaploReg^38^, the NHGRI/EBI GWAS Catalog (downloaded October 18, 2017)^39,40^, and UCSC known gene browser tables to identify overlap or proximity to neighbouring genes. For each associated SNP, we identified overlap with HaploReg components such as dbSNP functional annotation (5′-UTR, 3′-UTR, intergenic, nonsynonymous, synonymous), RoadmapEpigenomics epigenetic elements (promoter, enhancer, and DNase marks), and eQTL functional annotations across moderate-high LD variants (per-signal summary: Supplementary Worksheet S1; per-SNP summaries: Supplementary Worksheets S2 and S3).

Top variants associated with peach allergy lay within upstream regions of three *HLA* genes (*HLA-DQA1, HLA-DRB5, HLA-DRB1*), with 94% of the top peach SNPs (43/46 SNPs *r*^2^ > 0.8) overlapping epigenomic annotations (Supplementary Worksheet S2: 7 promoter marks, 32 enhancers, 23 TFBS), and 31 SNPs associated with gene expression in HaploReg (min. P~6.3 × 10^−47^; associated genes: *HLA-DRB6, HLA-DQA*2*, HLA-DRB1, HLA-DQB1, SKIV*2*L, SEC. 14L3*). Based on that observation, we examined SNPs that had at least moderate LD with the top associated peach allergy SNP (rs28359884) for overlap with eQTL variants identified in both European ancestry tissue samples (GTExPortal version 6p)^41^ and Japanese ancestry peripheral blood mononuclear cell (PBMC) samples (HGVD: Human Genetic Variation Database)^42,43^. That analysis identified significant GTExPortal + HGVD eQTL SNPs for two HLA genes (Supplementary Worksheets S4 and S5), HLA-DQA2 (min.*P*_*HLA-DQA*2*/GTEx*_ = 2.3 × 10^−56^, min.*P*_*HLA-DQA*2*/HGVD*_ = 1.3 × 10^−18^) and HLA-DRB5(min.*P*_*HLA-DRB5/GTEx*_ = 2.6 × 10^−29^, min.*P*_*HLA-DRB5/HGVD*_ = 1.8 × 10^−62^).

For shrimp allergy, top variants resided within a similar genomic HLA region encompassing six HLA class II genes (*HLA-DQA1, HLA-DRB5, HLA-DRB1, HLA-DQB1, HLA-DQA*2*, HLA-DRA*), with 60% of high LD SNPs (18/30 SNPs *r*^2^ > 0.8) overlapping potential regulatory elements (Supplementary Worksheets S3: 5 promoter marks, 16 enhancers, 2 DHS, 7 TFBS). Although only a single high LD variant (rs2760995) was an eQTL listed in HaploReg, analysis of moderate LD variants identified 18 SNPs associated with expression of one or more of four HLA genes (*HLA-DQA1*, *HLA-DQA*2, *HLA-DQB*2, and/or *HLA-DRA*) in both GTExPortal and HGVD (Supplementary Worksheets S4 and S6). Within 413 moderate LD SNPs, 162 SNPs were significantly associated with those genes’ expression in GTExPortal data, with *HLA-DQA*2 associated SNPs displaying the largest number of SNPs and highest significance levels across shrimp-allergy, GTExPortal, and HGVD eQTL phenotypic analyses (Supplementary Worksheet S4; min.*P*_*HLA-DQA*2*/GTEx*_ = 1.3 × 10^−51^, min.*P*_*HLA-DQA*2*/HGVD*_ = 5.3 × 10^−28^).

### Two-field HLA allele imputation analysis

Food-allergy is also considered to be related to an ability of particular HLA alleles to present exogenous food acquired peptides. Therefore, to better understand the underlying nature of the peach and shrimp allergy associations, we performed imputation of HLA alleles using the SNP genotype data (see Methods). To confirm the quality of the HLA imputation results, 126 samples with various representative HLA haplotypes were HLA typed by PCR-SSOP method. There was >99% concordance across both *HLA-DRB1* and *HLA-DQB1* genes between imputed and HLA typing results. To analyse the HLA data, we performed a Relative Predispositional Effects (RPE) analysis^44^, which performs a stepwise removal of top effect HLA alleles from cases and controls and then re-evaluates the significance of remaining alleles (see Methods)^45^. In that fashion, RPE attempts to account for the impact of strong effect alleles that may shift the distribution of other allele’s frequencies in cases away from those observed in the controls.

RPE analysis of the imputed HLA alleles for peach allergy identified four *HLA* alleles in *HLA-DRB1* (09:01, 14:05, 15:01, and 15:02) and *HLA-DQB1* (03:03, 06:02, 05:03 and 06:01) and two alleles in *HLA-DPB1* (14:01 and 09:01) (RPE summary in Table 2; RPE allele results in Table 3) as predisposing (PRE) alleles. While the preliminary stage of peach RPE analysis also identified alleles with protective effects (PRO), their significance decreased at each RPE step, suggesting that their presence was related to the strong PRE alleles (see DRB1*04:05 and *01:01*, DQB1*04:01 and *05:01* in Table 3 RPE step 0). Among the PRE alleles, HLA-DRB1*09:01 and HLA-DQB1*03:03 are shown to be most commonly expressed in Asian populations, among which Japanese have one of the highest frequencies (Supplementary Fig. S4a and S4b). RPE analysis of two locus *HLA-DRB1~HLA-DQB1* haplotypes showed significant associations for HLA-DRB1*09:01-HLA-DQB1*03:03, HLA-DRB1*14:05-HLA-DQB1*05:03, HLA-DRB1*15:01-HLA-DQB1*06:02, HLA-DRB1*15:02-HLA-DQB1*06:01 (Supplementary Table S2), while three locus *HLA-DRB1~HLA-DQB1~HLA-DPB1* haplotype analysis identified six haplotypes containing the significant two locus haplotypes in combination with three *HLA-DPB1* alleles (Supplementary Table S2). Among the associated two-locus *HLA* haplotypes, HLA-DRB1*09:01-HLA-DQB1*03:03 is shown to be exclusively expressed in Asian populations (Supplementary Fig. 4c).

**Table 2:** Relative Predispositional Effect (RPE) analysis locus summary.

Locus	RPE step	Peach allergy		Shrimp allergy	
		Significant alleles excluded	*P*-value	Significant alleles excluded	*P*-value
*HLA-DRB1*	0	none	4.27E-11	none	8.48E-15
	1	09:01	9.25E-08	04:05	1.58E-02
	2	09:01, 14:05	2.16E-05	04:05, 15:01	1.35E-01
	3	09:01, 14:05, 15:01	0.0028		
	4	09:01, 14:05, 15:01, 15:02	0.1384		
*HLA-DQB1*	0	none	2.69E-12	none	3.37E-16
	1	03:03	2.23E-08	04:01	1.12E-02
	2	03:03, 06:02	2.62E-06	04:01, 06:02	2.16E-01
	3	03:03, 06:02, 05:03	8.36E-04		
	4	03:03,06:02,05:03,06:01	0.2806		
*HLA-DPB1*	0	none	2.34E-03	none	5.16E-05
	1	14:01	0.0357	06:01	7.69E-03
	2	14:01, 09:01	0.1787	06:01, 14:01	0.063

*P*-value is for the 2xk chi-square test of significance of allergy status versus all HLA alleles at a locus after excluding alleles from both cases and control that were identified as “Significant alleles excluded” from the previous RPE step.

**Table 3:** Peach allergy: RPE analysis HLA allele summary.

*HLA-DRB1*	RPE step 0				RPE step 1				RPE step 2				RPE step 3			
	Allele frequency		P-value	OR	Allele frequency		P-value	OR	Allele frequency		P-value	OR	Allele frequency		P-value	OR
	Controls	Cases			Controls	Cases			Controls	Cases			Controls	Cases		
09:01	0.148	0.225	1.79E-07	1.67												
14:05	0.020	0.042	9.61E-05	2.21	0.023	0.055	9.98E-06	2.45								
15:01	0.077	0.111	2.16E-03	1.50	0.091	0.143	9.34E-05	1.68	0.093	0.152	2.69E-05	1.75				
15:02	0.107	0.134	0.033	1.29	0.125	0.173	2.22E-03	1.46	0.128	0.183	7.19E-04	1.52	0.141	0.216	4.82E-05	1.67
14:03	0.015	0.021	0.219	1.42	0.018	0.027	0.116	1.57	0.018	0.029	0.091	1.62	0.020	0.034	0.052	1.74
08:03	0.082	0.080	8.43E-01	0.97	0.097	0.103	0.627	1.08	0.099	0.109	0.468	1.12	0.109	0.129	0.222	1.21
04:05	0.131	0.077	9.19E-05	0.55	0.154	0.099	1.18E-03	0.61	0.157	0.105	2.67E-03	0.63	0.173	0.124	0.012	0.67
14:54	0.031	0.034	6.92E-01	1.09	0.037	0.044	4.05E-01	1.21	0.038	0.047	3.25E-01	1.25	0.042	0.055	0.195	1.34
12:02	0.020	0.008	0.043	0.41	0.023	0.011	0.073	0.45	0.024	0.011	0.087	0.47	0.026	0.013	0.120	0.50
01:01	0.060	0.034	8.61E-03	0.56	0.070	0.044	0.029	0.61	0.072	0.047	0.043	0.64	0.079	0.055	0.087	0.68
08:02	0.045	0.041	0.642	0.91	0.053	0.053	0.990	1.00	0.054	0.056	0.858	1.04	0.059	0.066	0.602	1.12
11:01	0.027	0.016	0.098	0.59	0.032	0.021	0.178	0.65	0.033	0.022	0.214	0.67	0.036	0.026	0.305	0.72
13:02	0.070	0.059	0.273	0.83	0.083	0.076	0.604	0.91	0.085	0.080	0.753	0.95	0.093	0.095	0.920	1.02
04:06	0.036	0.023	0.082	0.62	0.042	0.030	0.167	0.69	0.043	0.031	0.209	0.71	0.048	0.037	0.317	0.76
14:06	0.016	0.010	0.232	0.61	0.019	0.013	0.338	0.67	0.019	0.013	0.380	0.7	0.021	0.016	0.477	0.74
04:10	0.015	0.010	0.284	0.64	0.018	0.013	0.402	0.71	0.018	0.013	0.450	0.73	0.020	0.016	0.555	0.78
12:01	0.035	0.026	0.221	0.73	0.042	0.034	0.399	0.80	0.043	0.036	0.478	0.83	0.047	0.042	0.660	0.89
04:03	0.034	0.026	0.265	0.75	0.040	0.034	0.463	0.83	0.041	0.036	0.548	0.86	0.046	0.042	0.742	0.92
04:01	0.009	0.007	0.516	0.72	0.011	0.008	0.645	0.79								
16:02	0.008	0.010	0.703	1.17												
***HLA-DQB1***	**Controls**	**Cases**	**P-value**	**OR**	**Controls**	**Cases**	**P-value**	**OR**	**Controls**	**Cases**	**P-value**	**OR**	**Controls**	**Cases**	**P-value**	**OR**
03:03	0.160	0.238	1.41E-07	1.65												
06:02	0.072	0.104	2.05E-03	1.51	0.085	0.137	8.23E-05	1.70								
05:03	0.040	0.065	2.01E-03	1.66	0.048	0.085	1.77E-04	1.86	0.052	0.098	3.40E-05	1.99				
06:01	0.185	0.210	1.16E-01	1.17	0.220	0.275	4.11E-03	1.35	0.241	0.319	2.47E-04	1.48	0.254	0.354	1.28E-05	1.61
04:01	0.131	0.073	1.55E-05	0.52	0.156	0.095	2.71E-04	0.57	0.171	0.110	1.21E-03	0.60	0.180	0.122	4.03E-03	0.64
05:02	0.023	0.024	9.23E-01	1.03	0.027	0.031	0.635	1.14	0.030	0.036	0.484	1.21	0.032	0.040	0.371	1.27
05:01	0.063	0.033	1.99E-03	0.51	0.075	0.043	8.58E-03	0.56	0.082	0.050	1.85E-02	0.59	0.087	0.056	0.034	0.62
06:04	0.064	0.054	0.292	0.83	0.076	0.070	0.636	0.92	0.083	0.082	0.898	0.98	0.088	0.090	0.862	1.03
04:02	0.035	0.028	0.351	0.80	0.042	0.037	0.606	0.88	0.046	0.043	0.787	0.94	0.048	0.048	0.956	0.99
03:01	0.115	0.087	0.027	0.73	0.137	0.114	0.142	0.81	0.150	0.132	0.308	0.86	0.158	0.146	0.532	0.91
03:02	0.101	0.076	0.036	0.73	0.120	0.099	0.163	0.81	0.132	0.115	0.327	0.86	0.139	0.128	0.539	0.91
***HLA-DPB1***	**Controls**	**Cases**	**P-value**	**OR**	**Controls**	**Cases**	**P-value**	**OR**								
14:01	8.76E-03	0.021	2.27E-03	2.38												
09:01	0.095	0.125	0.014	1.35	0.096	0.128	0.010	1.37								
02:01	0.247	0.278	0.076	1.17	0.250	0.284	0.050	1.19								
04:02	0.100	0.074	0.037	0.73	0.100	0.076	0.045	0.74								
13:01	0.018	0.011	0.174	0.60	0.019	0.011	0.185	0.60								
02:02	0.028	0.021	0.262	0.73	0.028	0.021	0.281	0.74								
04:01	0.050	0.043	0.387	0.84	0.051	0.044	0.422	0.85								
14:01	8.76E-03	0.021	2.27E-03	2.38												
09:01	0.095	0.125	0.014	1.35	0.096	0.128	0.010	1.37								

RPE analysis of HLA alleles associated with shrimp allergy revealed two PRE alleles for *HLA-DRB1* (04:05 and 15:01), *HLA-DQB1* (04:01 and 06:02) and *HLA-DPB1* (06:01 and 14:01) (RPE summary in Table 2; RPE allele results in Table 4). As seen for the peach allergy, initially significant PRO alleles in RPE step 0 (HLA-DRB1*09:01, HLA-DQB1*03:03) were no longer significant after RPE adjustment for the top PRE alleles. Two locus *HLA* haplotype analysis (*HLA-DRB1~DQB1*) showed HLA-DRB1*04:05-HLA-DQB1*04:01 to be the single strongest HLA haplotype (Supplementary Table S3), accounting for most of the signal observed at the top meta-analysis SNP. Three locus *HLA* haplotype analysis (*HLA-DRB1-DQB1-DPB1*) did not identify a more significant association, as the signal observed at the major two locus haplotype was split into HLA-DRB1*04:05-HLA-DQB1*04:01-HLA-DPB1*05:01, HLA-DRB1*04:05-HLA-DQB1*04:01-HLA-DPB1*02*:01*, and HLA-DRB1*04:05-HLA-DQB1*04:01-HLA-DPB1*04:02 (Supplementary Table S3). Among the associated *HLA* alleles and haplotypes, HLA-DRB1*04:05, HLA-DQB1*04:01 and HLA-DRB1*04:05-HLA-DQB1*04:01 showed Asian-specific geographic distribution similar to that seen for the top peach HLA alleles and haplotype (Supplementary Fig. S5).

**Table 4:** Shrimp allergy: RPE analysis HLA allele summary.

*HLA-DRB1*	RPE step 0				RPE step 1			
	Allele frequency		P-value	OR	Allele frequency		P-value	OR
	Controls	Cases			Controls	Cases		
04:05	0.131	0.232	2.22E-16	2.00				
15:01	0.077	0.101	5.52E-03	1.35	0.089	0.132	4.56E-05	1.56
12:02	0.020	0.007	3.51E-03	0.34	0.023	0.009	0.011	0.39
16:02	0.008	0.002	0.025	0.23	0.010	0.003	0.043	0.26
08:02	0.045	0.048	0.667	1.07	0.052	0.062	0.197	1.22
04:01	0.009	0.012	0.396	1.29	0.010	0.015	0.205	1.46
09:01	0.148	0.112	1.28E-03	0.72	0.171	0.146	0.066	0.83
12:01	0.035	0.023	0.040	0.65	0.041	0.030	0.149	0.74
14:06	0.016	0.017	0.878	1.04	0.018	0.022	0.515	1.18
14:54	0.031	0.030	0.816	0.96	0.036	0.039	0.656	1.09
08:03	0.082	0.075	0.402	0.90	0.095	0.097	0.794	1.03
13:02	0.070	0.063	0.386	0.89	0.081	0.082	0.892	1.02
14:03	0.015	0.014	0.705	0.90	0.017	0.018	0.942	1.02
14:05	0.020	0.018	0.624	0.89	0.023	0.023	0.981	1.01
01:01	0.060	0.052	0.280	0.86	0.069	0.067	0.857	0.97
15:02	0.107	0.091	0.122	0.84	0.123	0.119	0.749	0.96
11:01	0.027	0.023	0.442	0.85	0.031	0.030	0.862	0.96
04:06	0.036	0.030	0.314	0.83	0.042	0.039	0.744	0.94
04:10	0.015	0.013	0.520	0.83	0.017	0.016	0.836	0.94
04:03	0.034	0.029	0.365	0.84	0.040	0.038	0.813	0.96
13:01	0.006	0.005	0.777	0.88	0.006	0.006	0.989	0.99
***HLA-DQB1***	**Controls**	**Cases**	**P-value**	**OR**	**Controls**	**Cases**	**P-value**	**OR**
04:01	0.131	0.229	2.22E-16	1.96				
06:02	0.072	0.096	2.74E-03	1.38	0.082	0.125	2.24E-05	1.59
03:03	0.160	0.123	1.49E-03	0.74	0.184	0.159	0.080	0.84
03:02	0.101	0.095	0.552	0.94	0.116	0.124	0.525	1.07
05:02	0.023	0.015	0.092	0.65	0.027	0.020	0.227	0.73
04:02	0.035	0.025	0.064	0.69	0.041	0.032	0.212	0.78
06:04	0.064	0.060	0.563	0.93	0.074	0.077	0.710	1.05
06:01	0.185	0.164	0.080	0.86	0.213	0.212	0.949	0.99
05:03	0.040	0.036	0.518	0.90	0.046	0.047	0.935	1.01
05:01	0.063	0.054	0.218	0.84	0.073	0.070	0.742	0.95
03:01	0.115	0.097	0.075	0.83	0.133	0.126	0.594	0.94
06:03	0.005	0.004	0.489	0.70				
***HLA-DPB1***	**Controls**	**Cases**	**P-value**	**OR**	**Controls**	**Cases**	**P-value**	**OR**
06:01	0.001	0.006	8.86E-05	5.24				
14:01	0.009	0.017	5.98E-03	1.97	0.009	0.017	5.63E-03	1.98
13:01	0.018	0.029	0.020	1.57	0.018	0.029	0.018	1.57
04:02	0.100	0.080	0.039	0.79	0.100	0.080	0.043	0.79
19:01	0.006	0.002	0.113	0.34	0.006	0.002	0.114	0.34
02:02	0.028	0.034	0.238	1.23	0.028	0.034	0.227	1.24
04:01	0.050	0.056	0.405	1.12	0.050	0.057	0.385	1.13
03:01	0.054	0.057	0.615	1.07	0.054	0.057	0.590	1.08
02:01	0.247	0.239	0.545	0.96	0.248	0.240	0.601	0.96
09:01	0.095	0.091	0.667	0.95	0.096	0.092	0.701	0.96
05:01	0.392	0.389	0.837	0.99	0.392	0.391	0.928	0.99

### Cross-reactivity for self-reported food reactions

**Figure 4:**
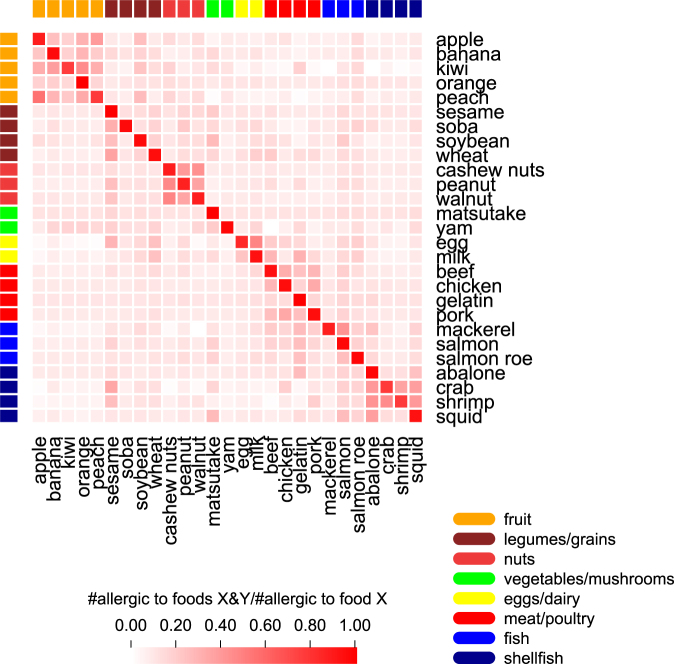
Cross-reactivity for different foods. Heatmap of the proportion of individuals who react to a food on the x-axis that also react to a food on the y-axis. Cross-reactivity between foods can particularly be seen within the larger groupings of fruit, nuts, and shellfish.

Previous food allergy reports have identified that individuals who react to one food sometimes react to other similar foods^5^ or even other environmental allergens^46^. Therefore, we examined the proportion of individuals reporting reactivity with one of the twenty-seven foods who also reported reactivity with another analyzed food. That analysis is summarized as a heatmap (Fig. 4), which shows three distinct clusters where individuals reported cross-reactivity between members of fruits, nuts, or shellfish food groups. We observed the highest cross-reactivity for the two foods for which we identified significant associations in this report, with 60.5% of individuals reporting a reaction to crab also reactive with shrimp (49.2% of shrimp sensitive subjects reported reaction with crab), and 70.6% of apple reactive subjects also reporting peach sensitivity (41.2% of peach reactive individuals reported sensitivity to apple).

We then examined the top peach and shrimp SNPs in the MHC region for associations with apple or crab, respectively. Both of the top peach and shrimp allergy associated SNPs were nominally significant in apple and crab reactive subjects (Top peach SNP: rs28359884, *P*_*apple*_ = 5.75 × 10^−6^; Top shrimp SNP: rs74995702, *P*_*crab*_ = 1.22 × 10^−3^). In addition, out of 18,679 SNPs in the region, about 50% of the top 100 peach or shrimp associated SNPs were also in the top 100 apple or crab associated variants (54 and 48 SNPs were in the top 100 peach and apple or shrimp and crab SNPs, respectively).

### Association analysis of previously reported SNPs

Recently, a GWAS of German children identified six SNPs (chr1:rs12123821; chr5:rs11949166; chr6:rs9273440, chr11:rs2212434; chr18:rs12964116, rs1243064) that were significantly associated with overall susceptibility to food allergy or individually to cows milk, hen’s egg, and/or peanuts^47^. Due to our study-design, we were not able to analyse all the Marenholz *et al*. SNPs for all three foods in our data, but we did analyse association with egg for those variants using the DISTMIX imputed GWAS statistics. Two of the six (rs12123821 and rs12964116) were monomorphic in East Asians, while the other four did not achieve even nominal significance (P < 0.05) in our dataset. We also calculated association statistics for peanut allergy using the regionally imputed data around the HLA for peanut allergy specific rs9273440 but also did not see a significant result (*P* = 0.1368). One also might expect that SNPs associated with overall susceptibility to food allergy would be associated across multiple other foods. Therefore, we examined all seven of our GWAS foods for association with chr1:rs12123821, chr5:rs11949166, chr11:rs2212434, chr18:rs12964116, and chr18:rs1243064. As noted, the filaggrin (*FLG*) locus SNP rs12123821, and rs12964116, one of two serpin peptidase inhibitor, clade B, member 7 (*SERPINB7*) locus SNPs, were monomorphic, and across the remaining three SNPs, we observed nominal significance in just one out of the seven foods for rs2212434 (*P*_*peach*_ = 0.01393) and rs1243064 (*P*_*peach*_ = 0.0390); we did not consider those as having passed multiple testing adjustment for the seven foods (*P* < 0.007). In addition, two variants had previously been identified in a GWAS by Hong *et al*. as associated with peanut allergy in U.S. children^26^. We found that one of the two SNPs was nominally associated with reactivity to peanuts in our dataset (rs7192: *P* = 0.00804; rs9275596: P = 0.0942). Potentially, the non-replication between our data and the Marenholz *et al*. study could be due to the low sample size in our data, case heterogeneity in our data due to use of self-reported food reactions rather than oral food challenge, or differences between children and adults with respect to food allergy aetiology.

## Discussion

In this study, we solicited 11011 adult females for self-reported allergy (SrFA) to any of 27 different foods and 21.5% reported sensitivity to one or more foods (Supplementary Table S1). That level is much higher than generally accepted estimates of 2–4% for the prevalence of food allergies (FA) based on the diagnostic gold standard of double-blind placebo controlled food challenge (DBPCFC), but not outside of the 10–30% estimates from previous studies that tabulated SrFA status^5,6,48–50^. Although one might presume that a large part of the difference between prevalence rates from SrFA and DBPCFC stems from patient subjectivity, some of the difference may be related to factors that augment allergic reactivity such as NSAID use, alcohol intake, exercise, menstruation cycle, and current pollen allergy season status^51^. For example, in birch pollen allergy patients, markers of gastrointestinal inflammation were significantly higher during the pollen allergy season than in the off-season^52^, suggesting that reactivity to oral food challenge (OFC) could depend upon seasonal fluctuation in inflammatory status. In addition, geographic regions may impart differential exposure to airborne allergens such as birch pollen (https://www.forestry.gov.uk/forestry/infd-8qnk35), which could lead to a larger local prevalence of FA to birch pollen related foods such as hazel nut, apple, peach, pear, etc. Also, exposure to potentially allergenic foods may differ due to regional consumption habits, with fruits and vegetables that are rare in some areas being very common in others and fish and shellfish consumption differing greatly between countries^53^. As an example, Chinese yam (Japanese yamaimo), which was the highest reported food in our study (Supplementary Table S1: 5.1%), is consumed widely in East Asia and is on the list of 25 foods for which the Japanese government requires labelling on food products^13^ but is not a common food in the United States or European countries. Since it is known to commonly elicit contact dermatitis when grated with bare hands^54^, the reported sensitivity in our study may relate to the external dermatological reaction rather than to a true FA response. Interestingly, although the SrFA rates observed in our study may seem high, the top foods largely mirror those identified as causative foods for new-onset FA and those responsible for accidental food ingestion by the Japanese national physician survey that was described in the Introduction^13^.

Seafood allergy is one of the common food allergies, with crustaceans representing 17.1% and 10.0% of new onset food allergies in Japan in 7–19 years and ≥20 years old age groups, respectively^13^. Interestingly, crustaceans are the second most reported cause of doctor’s visits due to accidental ingestion by Japanese adults, and shellfish are known to be the most common cause of anaphylaxis in adulthood in most Asian countries (Hong Kong (China), Singapore, and Thailand)^55–58^, US^59^ and Australia^60^ as well. In Japan, the most common types of edible shrimp are kuruma prawn (Marsupenaeus japonicus), White Pacific shrimp (Litopenaeus vannamei), black tiger (Penaeus monodon) and Japanese spiny lobster (Panulirus japonicas)^61^. Shrimp allergens are some of the most commonly researched among shellfish allergies^62^, with currently identified shrimp allergens including tropomyosin, arginine kinase, myosin light chain 1, myosin light chain 2, sarcoplasmic calcium-binding protein and troponin C and triosephosphate isomerase^61^. Among the shrimp allergens, tropomyosin is a pan-allergen that is considered to be the major shellfish allergen, and it has been reported that at least 80% of shrimp-allergy subjects respond to tropomyosin^30^. Cross-reactions between tropomyosins in the same class of shellfish are frequently reported, and it is known that the risk of reaction to a second species of crustacean is as high as 75%^63^. Besides that, the risk of dust mite allergy (Der p 10) is increased in those with allergy to crustaceans^63^. Binding assay of extracts of tropomyosin showed that roughly 75% of tropomyosin binds to shrimp-specific IgE from shrimp-allergic subjects^64^. Shrimp allergy is known to be mediated through the IgE mechanism, with the recognition by naïve CD4 + T cells of antigenic tropomyosin peptides presented by *HLA* class II molecules known to be the trigger for the IgE mediated hypersensitivity reaction^65,66^. Peptide binding assays on European patients showing positive reaction to brown shrimp tropomyosin identified 17 CD4 + T cell shrimp tropomyosin-derived epitopes restricted to DRB*01:01, DRB1*03:01, DRB1*04:01, DRB1*09:01, DQB1*02:01, DQB1*03:02 and DQB1*05:01. DRB1*04:05 and DQB1*04:01 identified in our analysis were not tested in that study as these two alleles are expressed only in Asian populations^65^. Interestingly, all shrimp epitope binding alleles tested in that study that are polymorphic in Asians (DRB*01:01, DRB1*04:01, DRB1*09:01, DQB1*03:02 and DQB1*05:01) were not significantly associated with shrimp allergy in our data. That observation suggests that additional mechanisms besides the presence of a particular allele with the capacity to bind a particular epitope are necessary to elicit an allergic response.

Peach (*Prunus persica*) is one of the most common fruits found to be related to allergy. The allergens most commonly found in peach include Pru P 1 (Bet v 1-homologous pathogenesis related protein 10 [PR 10]), Pru P 2 (thaumatin-like protein), Pru P 3 (lipid transfer protein), Pru P 4 (profilin), and Pru P 7 (antimicrobial peptide Gibberellin-regulated protein: GRB). Among the allergens, lipid transfer protein Pru P 3 is associated with severe peach allergy symptoms^67,68^, while on the other hand, Pru P 1 and Pru P 4 are mostly associated with milder forms of peach allergy (oral allergy symptoms)^32^. It has been suggested that the difference in severity of peach allergy might be related to the physical and chemical structure of the allergens, with Pru P 1 and Pru P 4 easily deactivated by the higher temperature and acidic environment in our gastrointestinal tract, while Pru P 3 is strongly heat and pepsin resistant^69^. Heat resistant allergens are usually unlikely to be outgrown and are known to be highly allergenic. Interestingly, individuals with the severe form of peach allergy are at higher risk of developing a severe form of tomato allergy^70^, even though the similarity between peach and tomato lipid transfer protein is only 49%^67^. Other reported peach allergy cross-reactivity includes fennel and to a rarer extent rice allergy^71^. In addition, a recent Japanese study found that 20% of 100 fruit-sensitive FA patients lacked srIgE reactivity to common birch tree Bet v 1 homologs or profilin, and that 65% of those lacked reactivity with lipid transfer proteins but did react with GRB^72^. Surprisingly, a majority of GRB reactive patients (84.6%) required some augmenting factor such as NSAID or exercise to develop an allergic response, suggesting that for particular antigens, food challenge by itself may not suffice to diagnose FA.

In this study, HLA-DRB1*09:01-HLA-DQB1*03:03 was found to be the top HLA susceptibility haplotype for peach allergy (*P* = 1.15E-07; *OR* = 1.68), but in contrast to shrimp allergy, three additional predisposing haplotypes with lower but comparable significance were identified by the RPE analysis (*P* ≤ 4.31E-05; *OR* range 1.68–2.46). Besides that, HLA-DRB1*09:01 is famously known to be the susceptibility *HLA* allele for Japanese rheumatoid arthritis (RA)^73^. A previous report has suggested a putative link between gut immunity and RA, with cross-reactive food antibodies (IgA, IgG and IgM) reported to be increased in the gut of RA patients^74^. Intervention studies have reported that decreasing animal protein and saturated fat intake and increasing fruit/vegetable, legume, and fish intake is associated with decreased levels of some markers of RA disease status^75,76^, which may relate to increased inflammatory status associated with the Western diet. That could suggest that food allergy may also contribute to increased inflammation in the gut and increased gut permeability that has been associated with RA^77,78^.

In addition to the food allergy associated HLA alleles and haplotypes, our analysis also found the top associated SNPs to be strongly associated in GTExPortal and HGVD data with the expression of various HLA genes. Among several HLA Class II genes for which SNPs were associated (Supplementary Worksheet S4), our analysis found only *HLA-DQA2* expression to be associated with both peach and shrimp allergy associated variants. *HLA-DQA2* and its beta-subunit *HLA-DQB2*, are paralogous genes to *HLA-DQA1 and HLA-DQB1* that are only lowly polymorphic but are highly conserved across both Old and New World primates^79^. Despite the strong conservation across species, *HLA-DQA2* was originally reported to be only lowly expressed and *HLA-DQB2* not expressed in lymphoblastoid cell lines^79,80^, and later *in vivo* footprinting assays found a lack of protein-DNA interactions in the same cell type^81^. However, a more recent report comparing the transcriptome of different dendritic cell types (DCs) identified strong expression of both *HLA-DQA2* and *HLA-DQB2* genes in Epidermal Langerhans Cells (LCs)^82^, in contrast to monocyte-derived, *CD1c*+, or plasmacytoid DCs, which lacked expression of the two genes. Further, that study identified cell surface expression of *HLA-DQA2*/*HLA-DQB2* heterodimers, and intriguingly, they also identified the presence of mixed *HLA-DQA2*/*HLA-DQB1* heterodimers and concluded that these genes’ expression in LCs could represent a new added level of complexity to the repertoire of antigens that could be presented by these cells. To confirm their results, we used FANTOM5 Phase 1 Cap Analysis of Gene Expression (CAGE) data^83,84^ to compare relative expression levels between the four *HLA-DQ* genes and found very different profiles in LCs compared to other lymphoid and dendritic cell-types (Supplementary Fig. S6). LCs displayed the highest CAGE expression levels of all four genes compared to other cell-types, with *HLA-DQB2* expression limited to just LCs and *HLA-DQA2* expression in LCs 2–7 times greater than that observed in the top non-LC cell-types. As a presumed subtype of dendritic cells, LCs are considered to be a type of antigen presenting cell that are found within the stratified squamous epithelial cells of the skin epidermis and oral mucosa^85^, as well as in gut-associated lymphoid tissues^86^. LCs have been described as central mediators of skin immunity^87^, have also been implicated in the pathogenesis of atopic dermatitis^88^, and are also a specific target for new allergen immunotherapies^89^. Since LCs appear to express the related *HLA-DQ* genes at higher levels than found in other cell-types, we suggest that LCs should be a target for future eQTL analyses.

In addition to the concerns noted above with respect to the inaccuracy of SrFA based studies, our questionnaire also did not query the subjects for the timeframe during which they had acquired their FA, whether they were responding to current or also childhood FA, or solicit them for whether their FA was of an immediate or delayed type response. Because of those concerns, we expect that cases in our analysis would represent a heterogeneous group. Some of that could possibly be captured by defining cases as those that experienced severe symptoms, namely loss of consciousness and/or anaphylaxis, but lack of power with such a small sample led us to combine the mild and severe responders into a combined case group. A larger sample set with a more comprehensive FA related questionnaire and follow-up with skin prick tests, srIgE, and DBPCFC in positive responders would likely lead to a finer understanding of whether HLA haplotypes and/or eQTL variants are responsible for different aspects of food allergy aetiology.

## Conclusion

This study identified variants in the HLA region that were associated with self-reported peach or shrimp allergy in a Japanese population sample. We found both shrimp and peach allergy to be associated with variants that are eQTLs for *HLA-DQA2* gene expression as well as associated with specific *HLA-DRB1* and *HLA-DQB1* alleles. However, of HLA alleles that were previously investigated for the capacity to bind shrimp epitopes^65^, none were associated in our dataset. Taken together, that may suggest that the genetics of food allergy is more complicated than the mere presence of a particular HLA allele that has a greater capacity to bind a certain food-antigen derived epitope, and that modulation of the expression of related HLA genes by genetic variants could play a role in the aetiology of certain food allergies. The general restriction of *HLA-DQA2* expression to LCs and the presence of LCs in various tissue-types that would be readily exposed to food during consumption (i.e., oral mucosa, gut associated lymphoid tissues) suggests that LCs may be a focal point for expanding our understanding of food allergy pathogenesis.

## Methods

### Subject, sample, and phenotype data collection

Subjects for this study were gathered by requesting users of MTI’s (http://www.mti.co.jp/eng/) “Luna Luna” women’s healthcare-related information website and apps to voluntarily participate in a study run by the MTI subsidiary EverGene to investigate the genetics of various human traits. Subjects were collected in two stages, denoted as LL01 and LL02, with DNA collected using saliva sampling kits (OraGene; DNA Genotek, Inc., Ottawa, Canada) and questionnaires soliciting trait information created using Survey Monkey (http://www.surveymonkey.com) and filled-out by subjects online. In total, we acquired 11379 subjects for analysis (LL01 = 5751, LL02 = 5628). The study design, including the consent form, general questionnaire topics, and genotyping, was approved by the Institutional Review Board at the Tsukuba International Clinical Pharmacology Clinic. The study was executed in accordance with applicable regulations and guidelines, and written informed consent was obtained from each patient for sample collection, genotyping, trait questionnaire, and trait analysis using genome-wide association study analysis.

### Sample processing, genotyping and quality control

Saliva sample kits were processed by Takara Bio (Kusatsu, Shiga Prefecture, Japan). LL01 and LL02 stage sample plates were genotyped separately for each stage by Takara Bio on a custom East Asian specific Axiom array (EverGene1). The EverGene1 chip contains 607857 total variants, with most variants chosen from Axiom CHB-1 chip SNPs that had MAF ≥ 0.01 in 1000 Genomes Project Japanese ancestry samples and additional custom variants selected from those with known pathogenic or phenotypic associations. Each stage’s genotypes were divided into separate batches and called separately using Affymetrix Analysis Suite 1.1.0616. Within the samples genotyped during each stage, there were 329 duplicate samples that we used to calculate genotype concordance between the two stages for each SNP. For association analyses, we only included autosomal and chromosome X variants that fulfilled the following criteria in both stages: 1) >99% call-rate, 2) MAF ≥ 0.01, 3) HWE P-value > 1 × 10^−6^, and 4) concordance-rate > 90%. After applying those filters, there were 536506 variants, of which 2417 were insertion-deletion polymorphisms (INDEL). Across those QC + variants, the average concordance-rate was 99.85 ± 0.30% (mean ± SD).

### Principal component analysis (PCA)

We downloaded genotype data for 2504 samples from the 1000 Genomes Project Phase 3 sample populations (ftp://ftp-trace.ncbi.nih.gov/1000genomes/ftp/release/20130502/) to help identify any admixed samples in our Japanese sample dataset. We then performed LD-pruning across the 1000 Genomes and LL01 and LL02 (LL01/LL02) genotype datasets using PLINK2 v1.90p (release date 16 Aug 2016)^90,91^ with *r*^2^ < 0.2, which identified 121595 SNPs with no or low-LD. We performed a principal component analysis (PCA)^92^ using PLINK with the LD-pruned SNPs, and after one round of PCA (PCA1; Supplementary Fig. S1a and S1b), we identified a small number of samples that were outliers (n = 19) to the typically recognized East Asian cluster^93,94^. Overlap with other populations suggests some level of admixture with European, African, or South Asian ancestry. To reduce downstream biases, we removed the admixed samples and performed a second round of PCA with LL01/LL02 + 1000 G EAS samples to help identify overlap of clustered samples with known East Asian sub-groups (PCA2; Supplementary Fig. 1c). After that, we performed a third round of PCA with just those LL01/LL02 samples so that the top PCs would reflect the main genetic axes of East Asian and Japanese population structure inherent to our population samples (PCA3; Supplementary Fig. 1d).

### Identification of duplicated samples

Using the same LD-pruned SNP data, we performed identify-by-descent (IBD) analysis using PLINK2 ver. 1.90p’s to identify potential duplicated samples. For eleven pairs of samples that appeared to be duplicate samples (PI_HAT > 0.8), we removed one from each pair from downstream analyses.

### Definition of food allergy cases and controls

Food allergy questionnaire items solicited as to whether an individual had either mild allergic reactions (“itching, hives, swelling of the lips and eyelids, vomiting”) or severe allergic reactions (“consciousness disorder, anaphylactic shock, such as a drop in blood pressure”) to a particular food. We coded food allergy cases as those that provided an affirmative response in either of the two response groups. For all food allergies examined, we used as controls 8350 individuals (LL01 = 4225; LL02 = 4125) that had no affirmative responses for either mild or severe food allergies. Within subjects who passed our sample and genotype QC procedures, there were 11011 samples who also responded to our food allergy questionnaire items and who were included in the GWAS analyses.

### Statistical analysis and genotype imputation

Basic data management, statistical analyses, and plotting were performed using the R 3.4.1 statistical programming environment^95^. Import and export of Microsoft Excel formatted data was done using the R package XLConnect^96^. We performed the primary association analysis using PLINK2’s logistic regression analysis method in an additive test for allelic association, with PC1 and PC2 from the PCA3 included as covariates. For seven foods that had more than 100 samples in both LL01 and LL02 stages, we utilized a bi-directional “discovery-evaluation” GWAS strategy^36^. Using that strategy, we used both sample sets simultaneously as discovery and evaluation and defined candidate SNPs as genotyped SNPs that fulfilled *P*_*discovery*_ ≤ 1 × 10^−4^ in one sampleset and *P*_evaluation_ < 0.05 and Benjamini-Hochberg FDR < 0.2 in the other sample set (Flow-chart: Supplementary Fig. S3). Meta-analysis statistics were calculated for each SNP using the inverse-variance weighting method^97^ to pool LL01 and LL02 samplesets’ beta-coefficients and standard errors as input. Based on the effective independent SNP count (M_E_) for a similarly sized array platform and the JPT population^37^, genotyped SNPs identified from the discovery-evaluation strategy were then called as nominally associated if they achieved a single GWAS *P*-value cut-off of *P*_meta_ < 1.21 × 10^−7^ (0.05/411,521). Those passing a multiple-testing adjusted *P*-value cut-off of *P*_meta_ < 4.4 × 10^−9^ (*P*_meta_ < 1.21 × 10^−7^/27 foods) were then considered as strongly associated. For producing the Manhattan plot (Fig. 1), we used the DISTMIX program^98^ and 1000 Genomes Project Phase 1 Release 3^99^ SNP correlation data to perform genome-wide imputation using the meta-analysis summary statistics; a manual population weight file was used to reflect a predominately Japanese population sample with slight proportions of other East Asian samples (JPT = 0.95, CHB = 0.04, CHS = 0.01). Output was filtered for SNPs that had DISTMIX info values greater than 0.8.

For two foods with SNPs achieving the significance cut-offs, we performed regional genotype imputation around associated variants by pre-phasing the LL01/LL02 genotyping dataset with EAGLE 2.3^100,101^ and then imputing using BEAGLE 4.1^102^ with the 1000 G Phase 3 reference haplotypes^103^. We imputed SNPs within 2 Mb of each associated signal, and then performed logistic regression analysis conditioning on the top imputed variant to better understand the structure of the association signal and identify un-genotyped top associated variants in each signal that could be candidate causal variants. Compared to BEAGLE imputed data, DISTMIX displayed inflated significance for top shrimp SNPs and depressed significance for associated peach variants.

### Linkage disequilibrium (LD) statistics

For sorting and ranking SNPs in LD with a top associated variant, we report two different measures. First, we report the common LD *r*^2^ measure produced using PLINK’s –r2 command, which calculates correlation of alternate allele dosage at two SNPs. Second, we calculated a simple metric, which we refer to as *r*^*2*^_*equiv*_, of how association statistics decrease after conditioning on a top SNP in a particular genomic region. For a single top SNP in a region, *r*^*2*^_*equiv*_ was calculated using −log_10_ transformed *P*-values of the top SNP A along with those of a tested SNP B before (B) and after conditioning (B|A). For test statistic *Z* = −log_10_(P), then *r*^*2*^_*equiv*_ = (*Z*_B_ − *Z*_B|A_)/*Z*_A._ Unless otherwise stated, moderate LD to a top SNP was defined as *r*^*2*^_*equiv*_ > 0.5 and high LD to a top SNP defined as *r*^*2*^_*equiv*_ > 0.8.

### *In silico* functional analysis of associated variants

We annotated variants using HaploReg 4.1^38^, which includes regulatory annotation for SNPs predicted to modify transcription factor motifs, DNase hypersensitivity sites (DHS), DNA methylation, evolutionary conservation scores (GERP), gene overlap, and eQTL annotations. HaploReg also includes annotation for overlap with previous GWAS data, but to include a more current dataset, we downloaded the NHGRI/EBI GWAS Catalog (dated October 18, 2017)^39,104^ and known gene tables from the UCSC Genome Browser web-site^105,106^. To examine overlap with transcription factor binding sites (TFBS), we created a .bed file for our variants, moved the genome-build coordinates from hg19 to hg38 using the UCSC liftOver tool, and then annotated them using the ReMap 2018 Annotation Tool^107^.

### eQTL analysis

We analysed eQTL data for both the European ancestry sample based GTExPortal version 6p^41,108^ and the Japanese ancestry sample based Human Genome Variation Database eQTL Release Version 8.1^43^. HGVD data was downloaded from their website (http://www.hgvd.genome.med.kyoto-u.ac.jp/eQTL/version.8.1/HGVDeQTL-V8_1-cis.tar.gz), and the GTExPortal significant cis-eQTL data file (GTEx_Analysis_v6p_eQTL.tar) was downloaded from https://gtexportal.org/home/datasets.

GTExPortal cis-eQTL data consists of two files each for 44 tissues: one file with a summary for each eQTL gene (*_Analysis.v6p.egenes.txt.gz) and the other with eQTL SNP-gene pairs (*_Analysis.v6p.signif_snpgene_pairs.txt.gz). The data as downloaded had been filtered by GTExPortal to included only significant SNP-gene pairs after permutation testing and application of a false-discovery rate (FDR < 0.05).

HGVD data consists of eQTL statistics files (sva.corrected.pheno.*.cis.gz) and annotation tables for SNPs (snpID.table) and genes (probeID.table). All were imported into R and merged into a single large table to use for analyses. While GTExPortal SNPs were already pre-filtered using the FDR, HGVD variants were not pre-filtered. To select SNPs that were determined by HGVD to account for some portion of the variance in a particular gene’s expression, we select HGVD SNPs that had at least *R*^*2*^ > 0.1 in HGVD’s PLINK^90,91^ based association analysis output.

SNP rsids used by HGVD and GTExPortal were updated to dbSNP147 when possible.

To identify SNPs associated with peach or shrimp food-allergy as well as with gene expression in GTExPortal and HGVD data, we selected moderate LD GWAS variants as those with either *r*^*2*^ > 0.5 or *r*^*2*^_*equiv*_ > 0.5 to the top GWAS SNP. Within each eQTL dataset, we calculated eQTL SNPs’ relative signal strength with respect to the minimum *P*-value at the top SNP for a particular gene as *RSS* = −log_10_(*P*_*SNP*_)/−log_10_(*P*_*Gene.min*_). We then labelled the moderate LD GWAS SNPs for whether they were tentative eQTL SNPs with *RSS*_*HGVD*_ > 0.2 or *max.RSS*_*GTEx*_ > 0.2 (*max.RSS*_*GTEx*_ is the maximum *RSS* value across all associated tissues for a particular SNP-gene pair). That filter is meant to select variants that have relatively strong evidence for being an eQTL compared to the larger set of variants in the GTExPortal and HGVD output. The final set of candidate eQTL regulated genes for each food allergy (Supplementary Worksheet S4) are genes for which at least one SNP was associated with its expression in both eQTL databases: (*r*^*2*^ > 0.5 or *r*^*2*^_*equiv*_ > 0.5) & *RSS*_*HGVD*_ > 0.2 & *RSS*_*GTEx*_ > 0.2. eQTL data shown in Supplementary Worksheets S5 and S6 is the larger set of gene-SNP pairs for which (*r*^*2*^ > 0.5 or *r*^*2*^_*equiv*_ > 0.5) & (*RSS*_*HGVD*_ > 0.2 | *RSS*_*GTEx*_ > 0.2); for GTEx Portal data, we filtered for data from higher confidence genes with one or more SNPs associated in a large number of tissue samples (≥10 tissue samples). One caveat for this analysis is that while GTExPortal eQTLs represents a rather comprehensive set of common variants, the HGVD data was based on micro-array genotyping with about 1.4 million analysed SNPs, and therefore, HGVD is missing data at about 75% of positions.

### HLA imputation

We extracted genotype data for EverGene1 SNPs between base pairs 25,759,242 and 33,534,827 (GRCh37/hg19 coordinates) on chromosome 6 and then performed *HLA* imputation for *HLA DRB1, DQB1*, and *DPB1* using the HIBAG R package^109^ along with Japanese specific HLA classifier data that we previously published^110^. Post-imputation quality control (Call Threshold, CT > 0.5) was applied to the imputed *HLA* alleles. To analyse concordance between imputed and laboratory determined *HLA* alleles, we selected 126 samples with representative imputed *HLA* alleles and *HLA* typing performed by an outside laboratory (Wakunaga Pharmaceutical Co., Ltd., Akitaka City, Hiroshima, Japan).

### HLA statistical analysis

Basic immunogenetic analyses of HLA loci and alleles were performed using the R BIGDAWG package^111^. To account for the impact of strong effect alleles, we implemented theses analyses in a Relative Predispositional Effect (RPE) framework^44,45^. RPE involves evaluation of a locus’ significance using a chi-square test of the 2xk contingency table of case/control status versus alleles, followed by a Pearson’s chi-square test of the 2 × 2 table of each individual allele’s significance if significance at a locus falls below a chosen cut-off (*P*_*locus*_ < 0.05). After the initial locus and allele evaluation (RPE step 0), the top effect allele (based on lowest *P*-value) was removed from both cases and controls, and then the locus and allele significance retested. That removal was then redone in a stepwise fashion, with subsequent top effect alleles added to the previous list for removal and re-evaluation until *P*_*locus*_ >  = 0.05. Alleles with expected counts less than five in cases or controls were automatically collapsed by the BIGDAWG software into a “binned” category for the analysis.

We performed initial 2-locus (*HLA-DRB1-DQB1*) or 3-locus (*HLA-DRB1-DQB1-DPB1*) haplotype analysis using BIGDAWG and then ran an analysis in an RPE framework using the estimated haplotypes. Only samples with no missing HLA alleles were used in the analysis, and *HLA* haplotypes with <1% frequency in either cases or controls were placed in the “binned” category.

### *HLA-DQ* gene expression in FANTOM5 data

We downloaded FANTOM5 CAGE RLE expression data for *HLA-DQA1*, *HLA-DQB1*, *HLA-DQA2*, *HLA-DQB2* genes from the Zenbu browser: (http://fantom.gsc.riken.jp/zenbu/gLyphs/#config = ONHzqgf2E5Xtmnpsh2gURB)^84,112^ for the “FANTOM5 CAGE Phase1 CTSS human tracks pooled filtered with 3 or more tags per library” track. We filtered the data for high-expressing tissue samples: those that had non-zero RLE values for a particular gene and then were in the upper-quartile of samples. We then extracted tissues that expressed two or more of the four genes for plotting in Supplementary Fig. S6.

### HLA allele and haplotype frequency data

We queried and downloaded frequency data for HLA alleles and haplotypes from The Allele Frequency Net Database (http://www.allelefrequencies.net/hla6006a.asp?)^113^ with the following URL command strings following the trailing question-mark:

HLA-DRB1*09:01

hla_allele1 = DRB1*09:01&hla_allele2 = DRB1*09:01

HLA-DRB1*04:05

hla_allele1 = DRB1*04:05&hla_allele2 = DRB1*04:05

HLA-DQB1*03:03

hla_allele1 = DQB1*03:03&hla_allele2 = DQB1*03:03

HLA-DQB1*04:01

hla_allele1 = DQB1*04:01&hla_allele2 = DQB1*04:01

HLA-DRB1*09:01-DQB1*03:03

hla_selection = DRB1*09:01-DQB1*03:03

HLA-DRB1*04:05-DQB1*04:01

hla_selection = DRB1*04:05-DQB1*04:01

### Figure plotting

Manhattan plots, regional association figures and other plots were produced using self-written R programs. HLA allele and haplotype frequency distribution maps were plotted using the R package *rworldmap* Ver.1.3-4^114^.

### Data availability

Original genotype data is not publicly available for the EverGene data due to strict consent requirements to protect subjects’ privacy. In its place, we provide files of genome-wide summary statistics for the genotyped and regionally imputed data for the two foods that possessed genome-wide significant association signals, with peach and shrimp allergy analyses provided as Supplementary Datasets S1 and S2, respectively. Files for the five foods lacking significant signals can be obtained upon request to the corresponding author.

## Electronic supplementary material

Supplementary Information

Supplementary Worksheets S1-S6

Dataset S1

Dataset S2
