# Supplementary material for: Genome-wide association study of self-reported food reactions in Japanese identifies shrimp and peach specific loci in the HLA-DR/DQ gene region
Source: Sci Rep. 2018 Jan 18;8:1069. doi: 10.1038/s41598-017-18241-w (PMC5773682; doi:10.1038/s41598-017-18241-w)
Supplement: Supplementary file 1 — Supplementary Information [file 41598_2017_18241_MOESM1_ESM.pdf]

Supplementary Information for:

Genome-wide association study of self-reported food reactions in Japanese identifies shrimp and peach specific loci in the *HLA-DR/DQ* gene region

Seik Soon Khor[1], Ryoko Morino[2], Kazuyuki Nakazono[3], Shigeo Kamitsuji[3], Masanori Akita[2], Maiko Kawajiri[2], Tatsuya Yamasaki[4], Azusa Kami[2], Yuria Hoshi[4], Asami Tada[2], Kenichi Ishikawa[2], Maaya Hine[5], Miki Kobayashi[5], Nami Kurume[5], Naoyuki Kamatani[3], Katsushi Tokunaga[1], and Todd A. Johnson[3,*]

[1]Department of Human Genetics, Graduate School of Medicine, The University of Tokyo, Tokyo, 113-0033, Japan

[2]EverGene Ltd., Shinjuku-ku, Tokyo, 163-1435, Japan

[3]StaGen Co., Ltd., Taito-ku, Tokyo, 111-0051, Japan

[4]Life Science Group, Healthcare Division, Department of Healthcare Business, MTI Ltd., Shinjuku-ku, Tokyo, 163-1435, Japan

[5]LunaLuna Division, Department of Healthcare Business, MTI Ltd., Shinjuku-ku, Tokyo, 163-1435, Japan

[*] Corresponding author

E-mail: todd.johnson@stagen.co.jp (TAJ)

Table of Contents

Supplementary Datasets 3

Dataset S1. Peach allergy GWAS summary statistics 3

Dataset S2. Shrimp allergy GWAS summary statistics 3

Supplementary Tables 4

Table S1. Demographic information for 27 foods 4

Table S2. RPE analysis of HLA haplotypes with peach allergy in Japanese population 4

Table S3. RPE analysis of HLA haplotypes with shrimp allergy in Japanese population 4

Supplementary Worksheets 5

Worksheet S1. Summary of GWAS statistics and annotations for association signals 5

Worksheet S2. GWAS statistics and annotation of top peach allergy associated SNPs 5

Worksheet S3. GWAS statistics and annotation of top shrimp allergy associated SNPs 5

Worksheet S4. Summary of top genes for eQTLs that overlap food allergy associated variants 5

Worksheet S5. Summary of eQTLs that overlap peach allergy associated SNPs 5

Worksheet S6. Summary of eQTLs that overlap shrimp allergy associated SNPs 5

Supplementary Figures 6

Figure S1. Population structure analysis 6

Figure S2. QQ plots of genome-wide meta-analysis association statistics 7

Figure S3. Study design flowchart 8

Figure S4. World distribution of top peach allergy associated HLA alleles and haplotype. 9

Figure S5. World distribution of top shrimp allergy associated HLA alleles and haplotype...10

Figure S6. HLA-DQA2 and HLA-DQB2 expression is largely restricted to Langerhans cells.11

Supplementary Datasets

Zipped archives of tabbed text files of GWAS summary statistics for genome-wide genotyped SNPs plus imputed variants in the chromosome 6 MHC region.

Common columns:

regional.analysis: Status flag with 0 if SNP data comes from GWAS micro-array data or 1 if SNP lies in imputed region.

CHR: Chromosomes numbered 1-23 for autosomes and chrX.

BP: Basepair position in hg19 coordinates

rsid: dbSNP147 rsID or concatenated ID with chrNN:BP.A2.A1

A1: Effect-allele and non-reference (alternate/ALT) allele in hg19

A2: hg19 reference/REF allele

A1.AF: Mean frequency of A1 allele across LL01 and LL02 study stages

P: Unconditioned P-value from linear (bust-size and dysmenorrhea) or logistic (menstrual fever) regression

BETA: Unconditioned beta-coefficient from linear (bust-size and dysmenorrhea) or logistic (menstrual fever) regression

SE: Unconditioned beta-coefficient standard error (SE) from linear (bust-size and dysmenorrhea) or logistic (menstrual fever) regression

Dataset S1. Peach allergy GWAS summary statistics

File: KhorSS_Supplementary_DatasetS1.tds

Dataset S2. Shrimp allergy GWAS summary statistics

File: KhorSS_Supplementary_DatasetS2.tds

Supplementary Tables

Table S1. Demographic information for 27 foods

LL01 control ct.=4225; LL02 control ct.=4125; Total control ct.=8350. Controls' mean age(SD)=32.37(6.7)

		Number of cases				
Food group	Food	LL01	LL02	Total ct.	Cases mean age(SD)	Included in GWAS analysis
Fruit	Apple	99	88	187	31.88(6.61)	
	Banana	75	75	150	32.33(7.23)	
	Kiwi	271	274	545	32.44(6.56)	*
	Orange	29	34	63	31.00(5.91)	
	Peach	158	162	320	32.44(6.72)	*
Legumes/grains	Sesame	11	6	17	35.06(7.11)	
	Soba	85	80	165	31.63(6.15)	
	Soybean	39	41	80	31.60(6.98)	
	Wheat	36	36	72	32.72(6.88)	
Nuts	Cashew nuts	21	21	42	32.21(6.54)	
	Peanut	22	31	53	31.62(6.65)	
	Walnut	20	28	48	31.58(5.96)	
Vegetables/mushrooms	Matsutake	6	2	8	31.00(6.21)	
	Chinese yam	307	256	563	33.03(6.65)	*
Eggs/dairy	Egg	124	147	271	31.66(6.47)	*
	Milk	56	49	105	33.24(7.24)	
Meat/poultry	Beef	18	17	35	33.17(6.10)	
	Chicken	13	14	27	33.00(6.40)	
	Gelatin	4	6	10	35.40(8.40)	
	Pork	11	17	28	35.18(5.73)	
Fish	Mackerel	128	124	252	34.14(6.67)	*
	Salmon	13	16	29	35.17(7.82)	
	Salmon roe	21	24	45	31.18(6.70)	
Shellfish	Abalone	16	23	39	34.46(6.30)	
	Crab	214	224	438	33.34(6.44)	*
	Shrimp	268	271	539	33.29(6.82)	*
	Squid	42	36	78	32.82(7.19)	

Last row of *DRB1-DQB1-DPB1* in Table S2 and S3 is not significant at the locus level but included for completeness.

Table S2. RPE analysis of HLA haplotypes with peach allergy in Japanese population

DRB1~DQB1	RPE.idx	Locus P-value	OR	Case Count	Total Count	Case Freq.	Control Count	Total Count	Control Freq.	RPE haplotype P-value
09:01~03:03	0	3.30E-12	1.684	138	610	0.226	2331	15756	0.148	1.15E-07
14:05~05:03	1	1.32E-08	2.458	26	472	0.055	311	13425	0.023	9.38E-06
15:01~06:02	2	7.55E-06	1.795	66	446	0.148	1157	13114	0.088	1.48E-05
15:02~06:01	3	1.69E-03	1.675	82	380	0.216	1687	11957	0.141	4.31E-05
**DRB1~DQB1~DPB1**										
14:05~05:03~05:01	0	1.84E-07	2.227	18	604	0.030	212	15580	0.014	9.70E-04
15:01~06:02~02:01	1	4.17E-06	1.765	38	586	0.065	581	15368	0.038	8.79E-04
09:01~03:03~05:01	2	8.19E-05	1.521	64	548	0.117	1183	14787	0.080	1.98E-03
15:02~06:01~09:01	3	8.04E-04	1.540	69	484	0.143	1326	13604	0.097	1.10E-03
09:01~03:03~02:01	4	0.0103	1.768	47	415	0.113	827	12278	0.067	2.82E-04
15:01~06:02~05:01	5	0.2171	1.742	19	368	0.052	347	11451	0.030	0.0201

Table S3. RPE analysis of HLA haplotypes with shrimp allergy in Japanese population

DRB1~DQB1	RPE.idx	Locus P-value	OR	Case Count	Total Count	Case Freq.	Control Count	Total Count	Control Freq.	RPE haplotype P-value
04:05~04:01	0	2.09E-14	1.990	233	1016	0.229	2050	15756	0.130	3.92E-19
15:01~06:02	1	0.039	1.552	98	783	0.125	1157	13706	0.084	8.07E-05
**DRB1~DQB1~DPB1**										
04:05~04:01~05:01	0	9.44E-10	1.836	135	992	0.136	1231	15580	0.079	2.33E-10
04:05~04:01~02:01	1	1.00E-03	2.118	32	857	0.037	258	14349	0.018	5.70E-05
04:05~04:01~04:02	2	0.081	1.658	29	825	0.035	303	14091	0.022	9.80E-03

Supplementary Worksheets

Available in KhorSS_SupplementaryWorksheets.xlsx

First four worksheets in file contain Legends with descriptions of individual columns in the following worksheets.

Worksheet S1. Summary of GWAS statistics and annotations for association signals

Descriptive information, aggregated statistics, and annotations of association signals.

Worksheet S2. GWAS statistics and annotation of top peach allergy associated SNPs

Descriptive information, statistics, and annotation of top peach allergy associated SNPs

Worksheet S3. GWAS statistics and annotation of top shrimp allergy associated SNPs

Descriptive information, statistics, and annotation of shrimp allergy associated SNPs

Worksheet S4. Summary of top genes for eQTLs that overlap food allergy associated variants

Significant SNP-gene eQTL associations from HGVD and GTExPortal were filtered for SNPs that were in at least moderate LD (r^2>0.5 | r^2_{equiv}>0.5) to either the top peach (rs28359884) or shrimp (rs74995702) associated SNP. Then, they were filtered for SNPs that we labelled as valid eQTLs in both HGVD and GTExPortal: 1) HGVD associations with HGVD *R2*>0.1 and *RSS*>0.2, 2) GTEx *max.RSS*>0.2. For each set, the table compares the mean β-coefficient of the peach or shrimp allergy logistic regression with those from the GTEx and HGVD eQTL analyses. Columns are grouped summarising across all SNPs or for those with both GTEx and HGVD eQTL values.

Worksheet S5. Summary of eQTLs that overlap peach allergy associated SNPs

Worksheet S6. Summary of eQTLs that overlap shrimp allergy associated SNPs

We overlapped eQTL SNPs for GTExPortal (*max.RSS*>0.2) and HGVD (HGVD *r2*>0.1 and HGVD *RSS*>0.2) with the SNPs that were in moderate LD to each peach or shrimp allergy associated locus' top SNP and show the eQTL gene, eQTL tissues, eQTL slope, and GWAS beta-coefficient. Both the slope and beta-coefficient measure effect of A1 (Alternate GRCh37/hg19) allele. Imputation was not performed by HGVD (all SNPs from Illumina Omni5Exome beadchip), so many of the SNPs that were associated in both the GWAS and GTEx Portal datasets are missing HGVD values at the top variants, while missing values for GTExPortal data are generally due to the pre-filtering that was applied to the original tables. Data shown is for: 1) genes that had at least one SNP that was an eQTL in ≥10 GTEx Portal Tissues or identified in HGVD (only a single tissue analysed), 2) only for HLA-genes.

Supplementary Figures

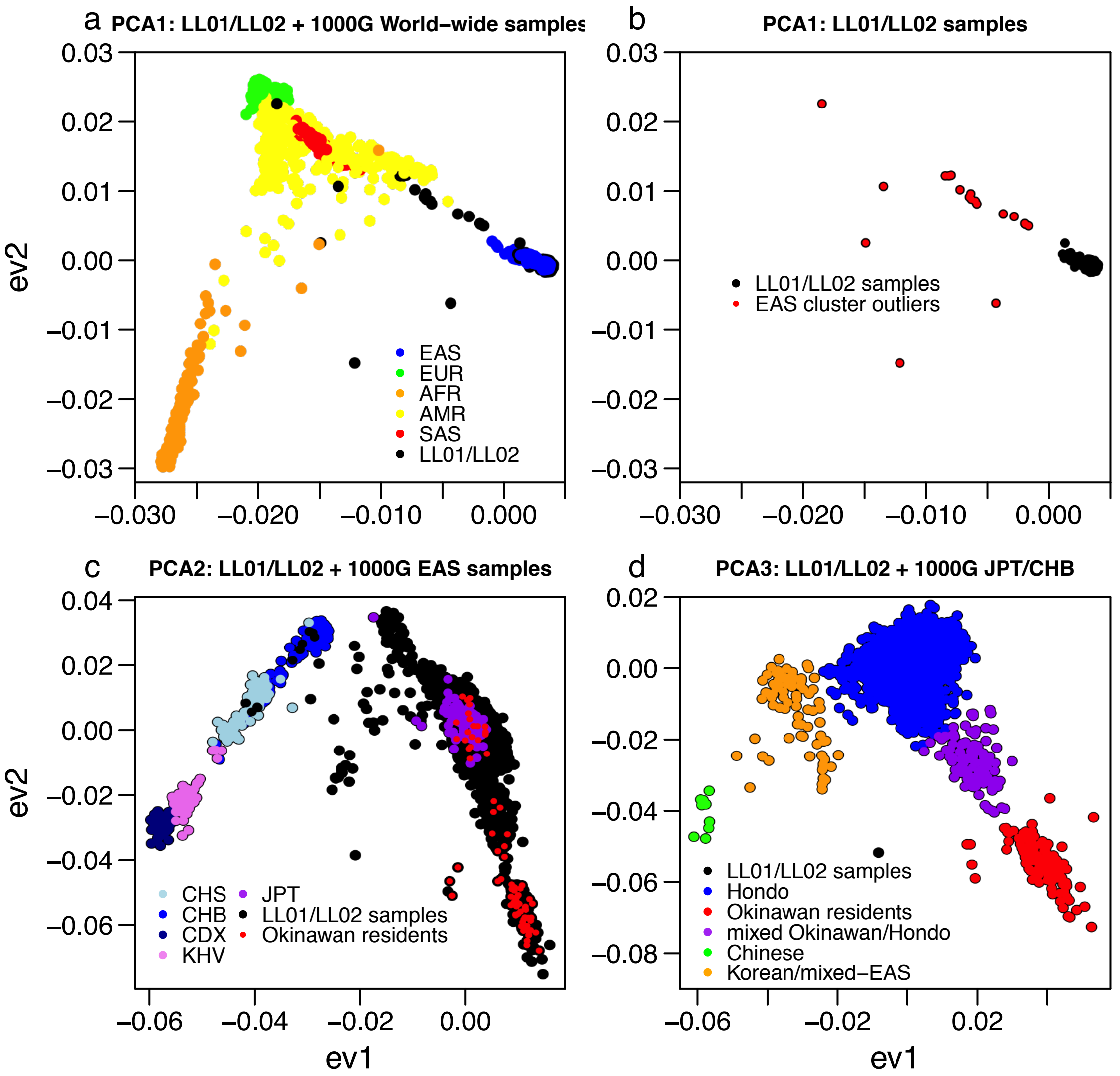

Figure S1. Population structure analysis

PCA was performed in three runs, with PCA1 combining LL01+LL02 and 1000G world-wide samples, PCA2 combining LL01+LL02 (minus non-Asian outliers) with 1000G Asian samples, and PCA3 combining LL01+LL02 with 1000G JPT/CHB samples. **Panel a)** PCA1, **Panel b)** PCA1 zoomed to LL01+LL02 samples, **Panel c)** PCA2, and **Panel d)** PCA3.

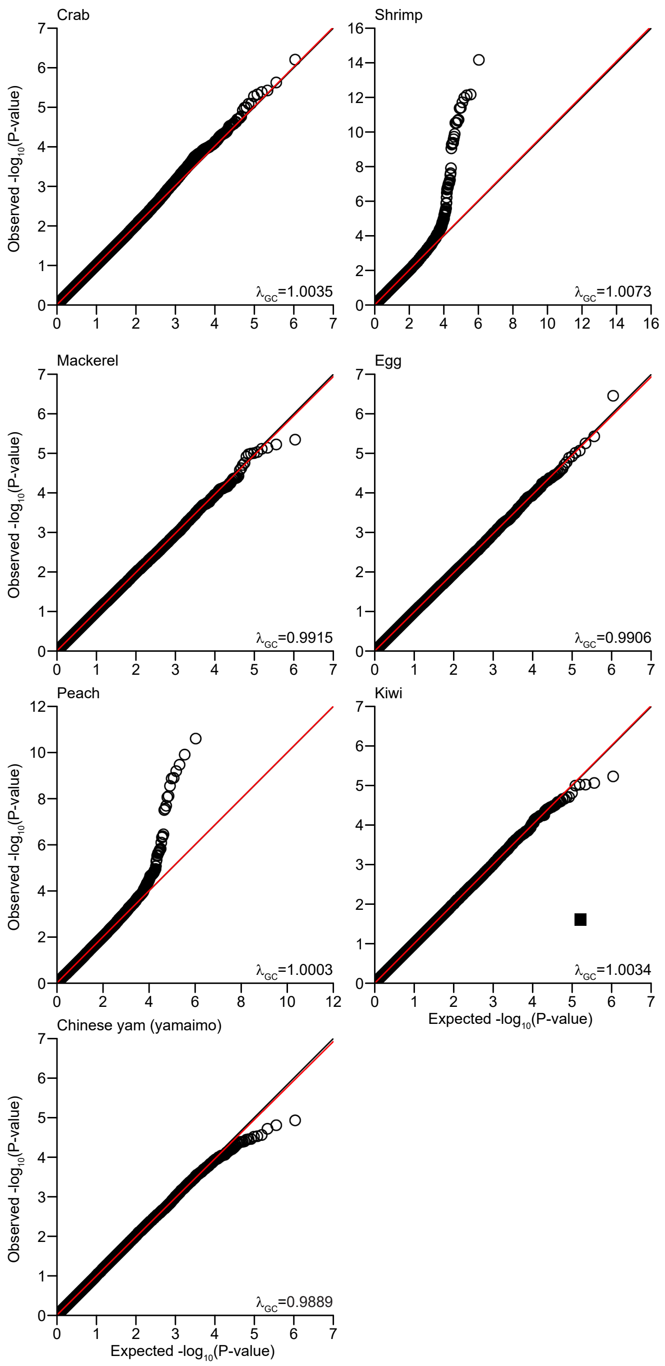

Figure S2. QQ plots of genome-wide meta-analysis association statistics

QQ plot of expected and observed $-\log_{10}(P_{meta})$ for genotyped GWAS statistics for seven foods with >100 samples in both LL01 and LL02 sample-sets. λ_{GC} was calculated as the median of the observed χ^2 test statistics divided by the median of test statistics assuming a uniform distribution. Black line has slope of 1.0. Red line has slope of λ_{GC}.

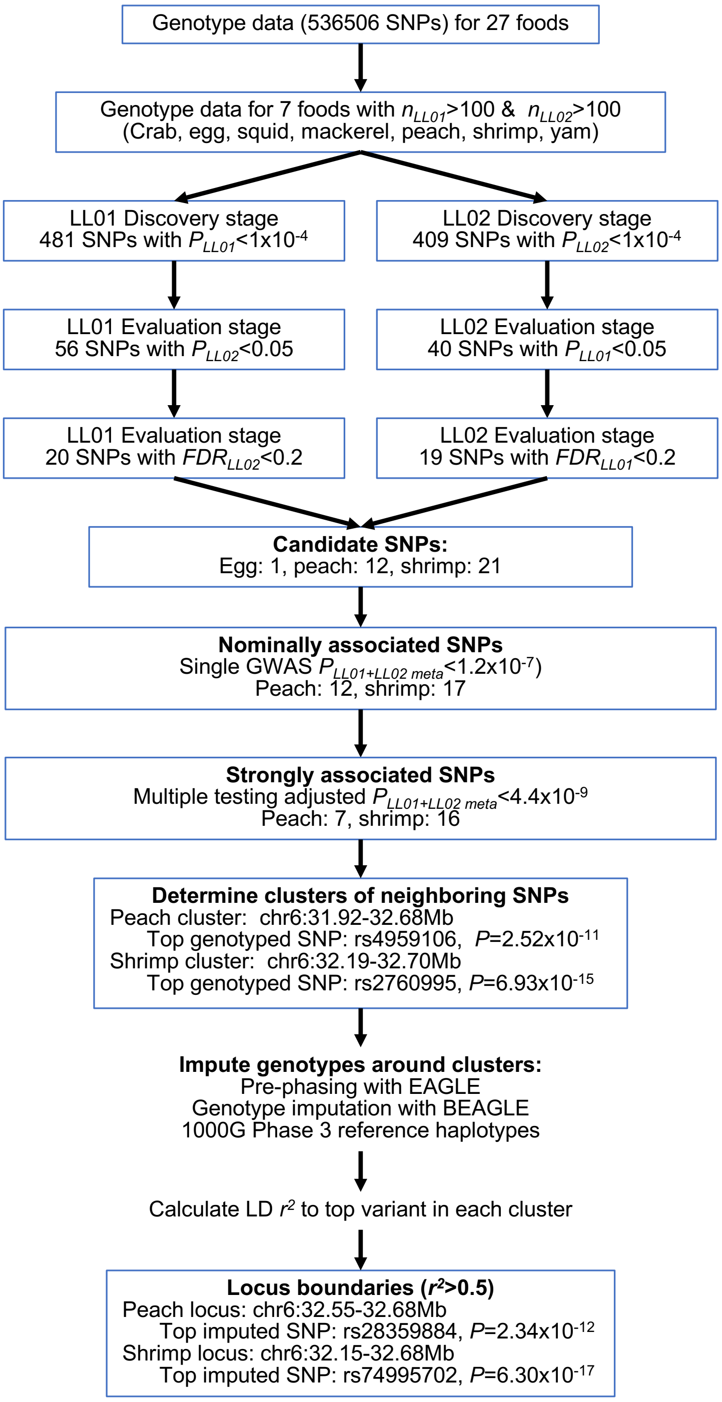

Figure S3. Study design flowchart

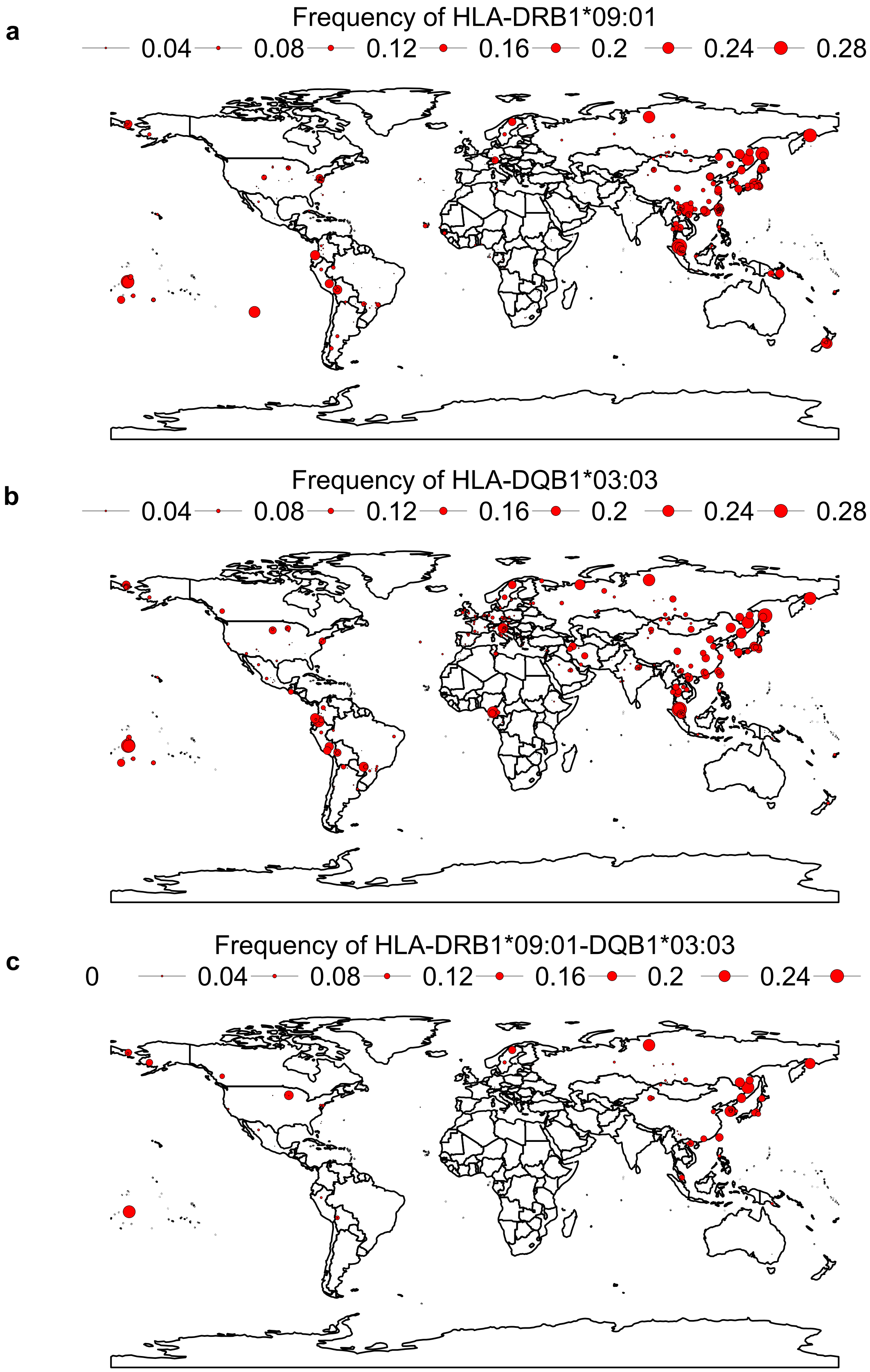

Figure S4. World distribution of top peach allergy associated HLA alleles and haplotype.
Plotted using the R *rworldmap* package Ver.1.3-4 with HLA allele and haplotype data from http://www.allelefrequencies.net. Population samples in the same location were sorted by frequency and jittered to allow better visualisation. **Panel a)** *HLA-DRB1*09:01*, **Panel b)** *HLA-DQB1*03:03*, and **Panel c)** *HLA-DRB1*09:01~HLA-DQB1*03:03*.

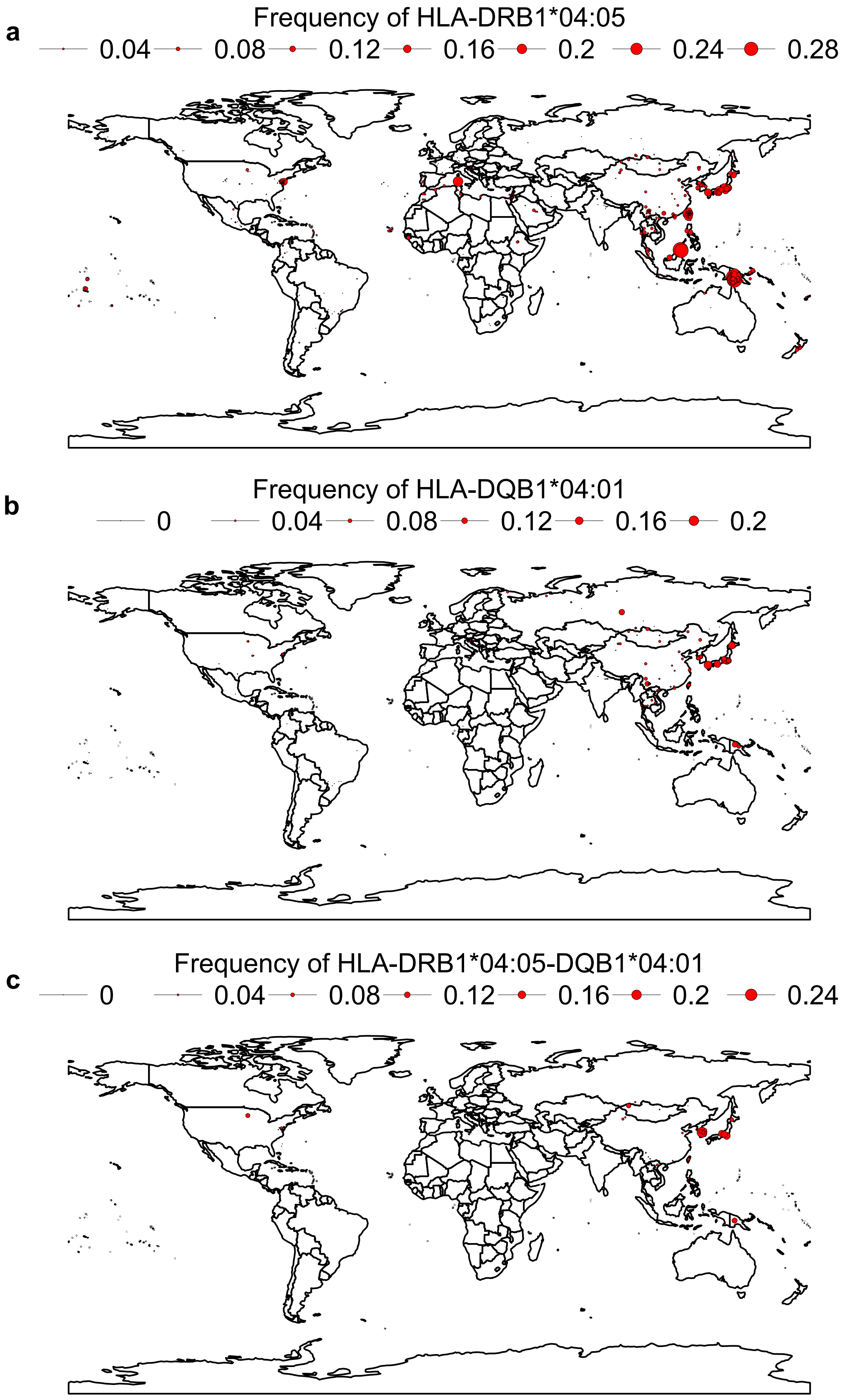

Figure S5. World distribution of top shrimp allergy associated HLA alleles and haplotype.

Plotted using the R *rworldmap* package Ver.1.3-4 with HLA allele and haplotype data from http://www.allelefrequencies.net: Population samples in the same location were sorted by frequency and jittered to allow better visualisation. **Panel a)** *HLA-DRB1*04:05*, **Panel b)** *HLA-DQB1*04:01*, and **Panel c)** *HLA-DRB1*04:05~HLA-DQB1*04:01*

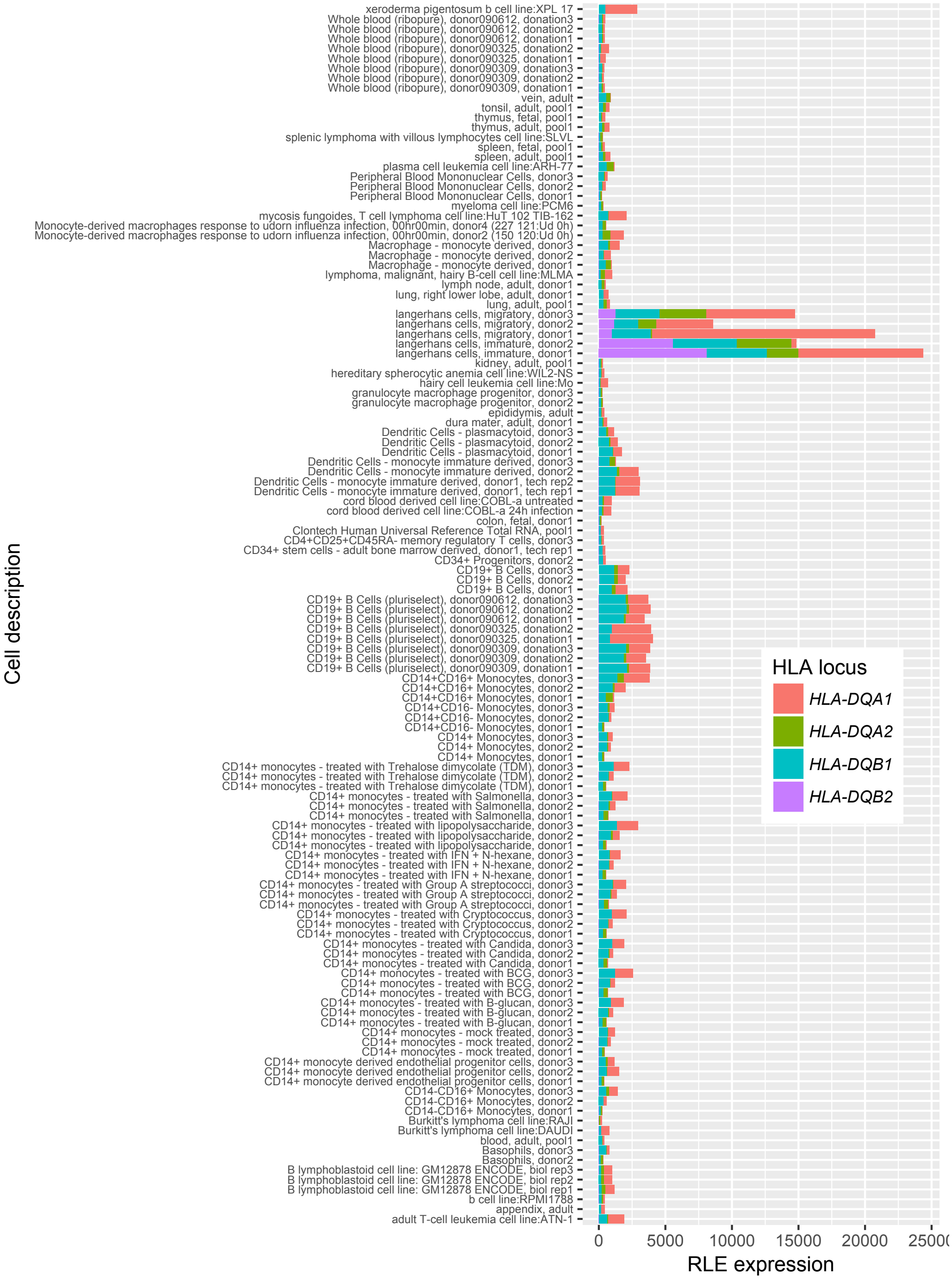

Figure S6. ***HLA-DQA2* and *HLA-DQB2* expression is largely restricted to Langerhans cells.**

FANTOM5 Phase 1 CAGE RLE expression values for *HLA-DQA1*, *HLA-DQB1, HLA-DQA2*, and *HLA-DQB2* were downloaded from http://fantom.gsc.riken.jp/zenbu/gLyphs/#config=ONHzqgf2E5Xtmnpsh2gURB and filtered for tissues that were in the upper-quartile of tissues expressing a particular gene (UQ calculated across tissue samples with RLE>0). The merged table of HLA gene vs. tissue expression was then further filtered for tissues that expressed at least two of the four genes. *HLA-DQB2* expression is only expressed in Langerhans cells, while *HLA-DQA2* expression is mostly restricted to Langerhans cells, with only limited expression in other cell types.
